# The alternative NF-κB RelB subunit is a novel critical sensor of lipid metabolism to promote tumor development

**DOI:** 10.1038/s41419-026-09064-7

**Published:** 2026-07-04

**Authors:** T. Becquard, C. Benatar, C. Bretot, B. Eluard, D. Bordereaux, L. Thirouard, A. Hammoutene, A. Montagne, F. Dingli, D. Loew, C. Ransy, M.-C Alves-Guerra, C. Caradeuc, N. Cagnard, M. Boissan, N. Giraud, F. Bouillaud, G. Bertho, V. Paradis, D. Chiron, R. Dentin, C. Prip-Buus, V. Baud

**Affiliations:** 1 Université Paris Cité, NF-κB, Differentiation and Cancer, Paris, France; 2 Université Paris Cité, CNRS, INSERM, Institut Necker Enfants Malades -INEM, Paris, France; 3 Nantes Université, Inserm, CNRS, Université d’Angers, Nantes, France; 4 Université Paris Cité, AP-HP. Nord, Department of Pathology, INSERM UMR 1149, FHU MOSAIC, SIRIC InsiTu, Beaujon Hospital, Clichy, France; 5 Institut Curie, PSL Research University, CurieCoreTech Mass Spectrometry Proteomics, Paris, France; 6 Université Paris Cité, CNRS, Inserm, Institut Cochin, Paris, France; 7 Université Paris Cité, UMR 8601 CNRS, Laboratoire de Chimie et Biochimie Pharmacologiques et Toxicologiques LCBPT, Paris, France; 8 Bio-informatic Platform, Université Paris Cité, INSERM US24/CNRS, Unité Mixte de Service (UMS) 3633, Paris, France; 9 Sorbonne Université, INSERM UMR_S 938, Centre de Recherche Saint-Antoine CRSA, Paris, France

**Keywords:** Cancer metabolism, Oncogenes

## Abstract

Cancer cells reprogram their metabolism to fulfill their high energetic demand. Lipid metabolism is most often reprogrammed for cancer cell survival and tumor development. The role of alternative oncogenic NF-κB/RelB subunit in the reprogramming of lipid metabolism in cancer is unknown. Here we report that RelB plays a central role at the crossroads of lipid storage and liberation of fatty acids from the lipid droplets to feed the fatty acid oxidation (FAO) and mitochondrial energetic metabolism. High RelB expression defines a subset of hepatocellular carcinoma (HCC) patients and cell lines with a peculiar gene expression profile enriched in lipid catabolic-related genes, including lipases. Functional studies revealed that high RelB activation controls the expression of major lipolytic lipases, including adipose triglyceride lipase (ATGL) and monoglyceride lipase (MAGL), and impacts on HCC cell survival, migration, and tumor development in vivo. Altogether, we uncovered that RelB is a central regulator of the lipid metabolism plasticity and an energy homeostasis sensor in cancer cells.

## Introduction

Cancer cells exhibit bioenergetics alterations in order to meet their high-energy requirement, and deregulation of cellular energetics has been recognized as one of the hallmarks of cancer [1]. Lipids function as essential building blocks for membranes, but also serve as fuel to drive energy-demanding processes and are regulators of numerous cellular functions [2]. Lipid metabolism is most often reprogrammed for cancer cell survival and tumor development [3] due to activated oncogenic pathways and environmental cues [4].

Aberrant NF-κB activity has been observed in many cancers, including both solid and hematopoietic malignancies, and NF-κB transcription factors are major players in the control of cancer cell proliferation and survival, as well as in the pathophysiology of numerous cancers [5–10]. The NF-κB transcription factor family consists of five members in mammals: RelA (p65), RelB, cRel (Rel), NF-κB1 (p50 and its precursor p105), and NF-κB2 (p52 and its precursor p100), and forms a collection of various homodimeric and heterodimeric complexes [11]. The activity of NF-κB is regulated by two main pathways. The first one, known as the canonical NF-κB activation pathway, mainly applies to RelA- and/or cRel-containing complexes [12]. The second one, the alternative NF-κB activation pathway, leads to the activation of RelB-containing dimers [7, 13, 14]. How the alternative NF-κB/RelB subunit may impact on lipid metabolism in cancer cells, and its connection with the pathogenic process of cancer cells and poor patient outcome is currently unknown.

In the study presented here, we reveal a role for the alternative NF-κB/RelB subunit in the plasticity of lipid metabolism and energy homeostasis in cancer cells. RelB defines a new subset of hepatocellular carcinoma (HCC) cell lines and HCC patients with poor prognosis, presenting a peculiar, distinct gene expression profile enriched in lipases and lipase-related genes. Functional studies revealed that RelB drives HCC cancer cells to oxidize fatty acids (FA) for mitochondrial activity at the expense of lipid storage in lipid droplets. RelB-dependent control of FA oxidation (FAO) is linked to Adipose Triglyceride Lipase (ATGL) and Monoglyceride Lipase (MAGL) expression and impacts on HCC cell survival, migration, and tumor development in vivo. Ultimately, we show that RelB positivity is associated with high protein expression of ATGL and low lipid droplet content in HCC patient samples. Altogether, our data highlight a previously unrecognized function for the alternative RelB NF-κB subunit as an energy sensor of cancer cell lipid metabolism and provide the framework for the development of new drugs targeting RelB to overcome resistance in HCC.

## Results

### RelB controls lipases expression in HCC cancer cells

**Fig. 1:**
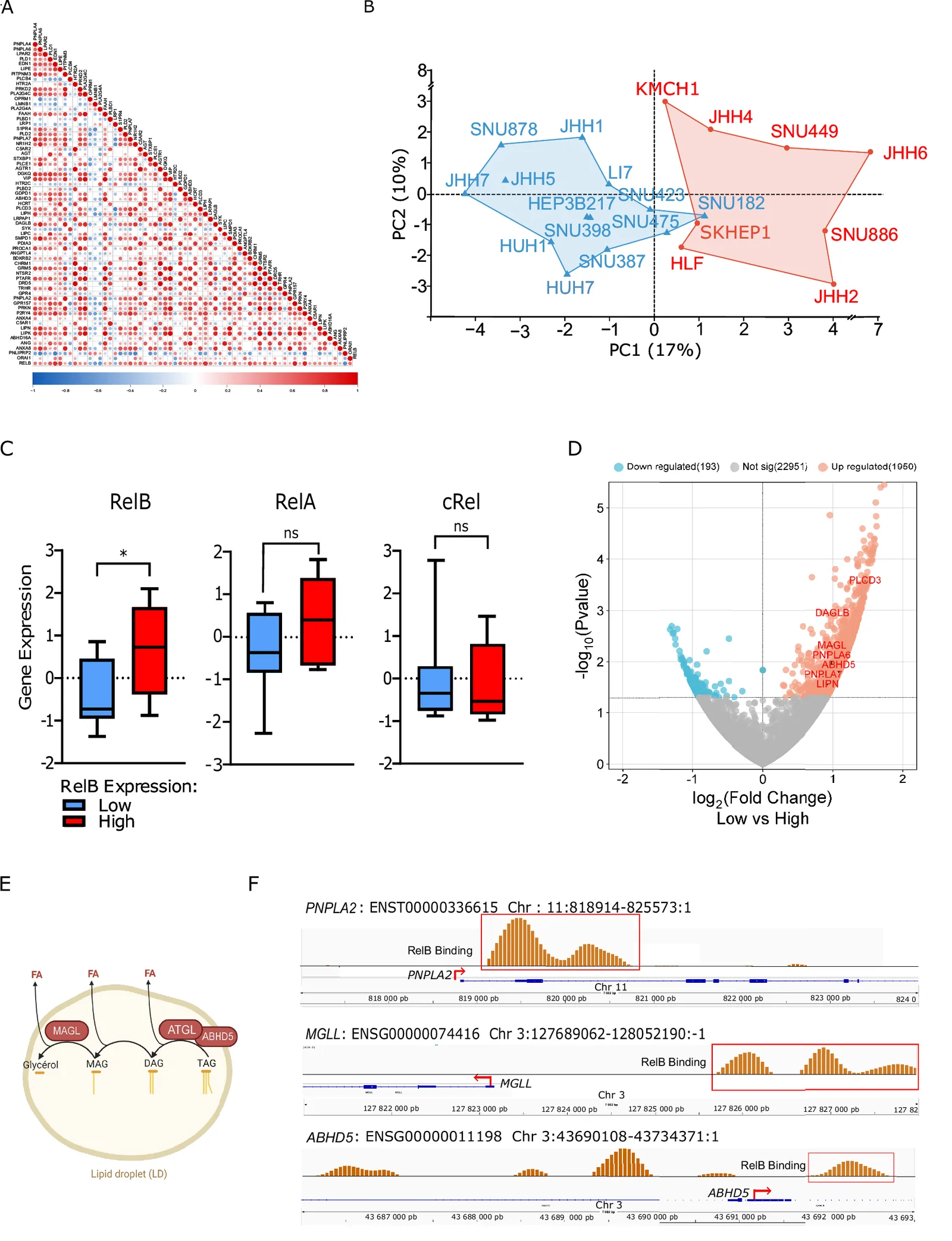
RelB controls lipases expression in HCC cancer cells. **A** Correlation matrix between mRNA expression levels of RelB and sixty-four lipases from the CCLE HCC cell lines dataset. Pearson correlation values are indicated. **B** Identification of two clusters in the CCLE HCC cell lines dataset (*n* = 21) by unsupervised learning *K*-means clustering, Python (V3.11.9). **C** Significant difference in RelB, but not RelA and cRel, expression between the two clusters as defined in (**B**). High-RelB vs low-RelB clusters are indicated in red and blue, respectively. *P*-values by unpaired *t*-test, *p < 0.05, error bars represent min and max values. **D** Volcano plot depicting the differences of gene expression between high-RelB and low-RelB clusters as defined in (**B**) (*x*-axis, log2 fold change; *y*-axis, -log10, *p* value > 0.05). *P* value by Fisher’s exact test, significance thresholds, *p* < 0.05. Several key lipases are highlighted in red. **E** Schematic representation of the two main lipases ATGL and MAGL, and the ATGL co-regulator ABHD5 involved in the release of FA from stored TAG in lipid droplets, the so-called lipolysis. ATGL Adipose Triglyceride Lipase, MAGL Monoacylglycerol Lipase, ABHD5 Hydrolase Domain-Containing Protein 5, FA fatty acids. **F** RelB recruitment to the promoter of *PNPLA2*, *MGLL,* and *ABHD5*. The RelB recruitment is indicated by bars and framed in red. Data from ChiP Atlas, GSE 55105. Visualization by IGV browser.

To assess how RelB may impact on the near-universal reprogramming of lipid metabolism in cancer cells, we developed different correlation matrixes using the Cancer Cell Lines Encyclopedia (CCLE) HCC cell lines dataset to evaluate the lipid metabolism features that correlated with RelB expression levels. A strong correlation was observed between RelB gene expression and a large set of lipases and lipase-related genes (Fig. 1A and Supplementary Table 1). Further, we wanted to confirm the association between RelB and lipases by a second approach. In a first step, using the unsupervised K-means clustering, the HCC cell lines were separated into two different subsets (Fig. 1B) that were significantly associated with *RelB* expression (Fig. 1C). To go one step further, we then cataloged genes whose expression was associated with high levels of RelB expression. Interestingly, the high-RelB cluster was characterized by an enrichment of lipases (Fig. 1D), thus further indicating the inter-relation between RelB and lipases. No significant correlation was observed between the high-RelB cluster of HCC cell lines and the expression of either *RelA* or *cRel*, the two canonical NF-κB subunits (Fig. 1C). As other control genes, *PPARA*, *PPARD,* and *PPARG* that are important regulators of lipid metabolism did not correlate with the high-RelB expression cluster of HCC cell lines (Supplementary Fig. 1A).

Further evidence for a direct role for RelB in regulating lipase transcription was obtained by chromatin IP (ChIP) analysis. RelB is efficiently recruited to the promoter of various lipases, including the central lipolytic lipases ATGL (*PNPLA2*), MAGL (*MGLL*), and the ATGL co-regulator ABHD5 for the release of FA from stored triacylglycerols (TAG) in lipid droplets (Fig. 1E, F and Supplementary Fig. 1B).

### RelB defines a new subset of HCC patients with a GEP enriched for lipase-related genes and associated with worse outcome

**Fig. 2:**
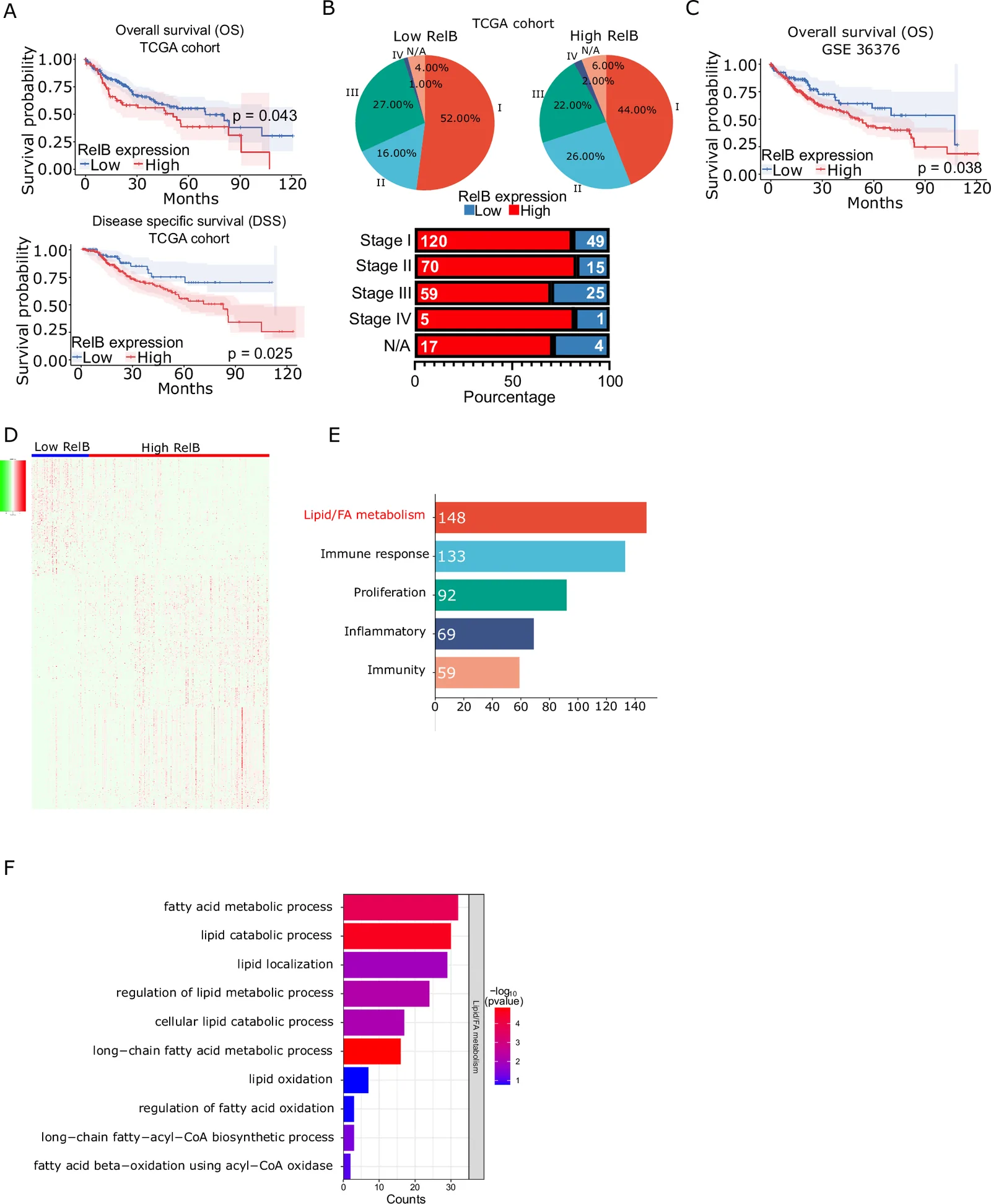
RelB defines a new subset of HCC patients with a GEP enriched for lipase-related genes and associated with worse outcome. **A** Optimized cutoff of RelB expression using Python (V3.11.9) identifies a subset of HCC patients associated with poor outcome. Kaplan Meir plots of disease specific survival (DSS) and overall survival (OS) according to RelB mRNA expression levels (LIHC-TCGA cohort, *n* = 365). *P* value by log-rank (Mantel-Cox) test. **B** Top: prevalence of the indicated HCC stages according to the AJCC 7th classification within the high-RelB and low-RelB clusters as defined in (**A**). Bottom: Prevalence of RelB expression as defined in (**A**) within the indicated HCC stages as defined by the AJCC 7th classification. **C** Patient outcome according to RelB expression clusters in an independent validation cohort (GSE 36376, *n* = 239). *P* value by log-rank (Mantel–Cox) test. **D** Hierarchical clustering of the HCC patients from the LIHC-TCGA cohort (*n* = 365) with the 872 genes identified by RNAseq based on RelB expression. Lines and columns of the heatmap correspond to genes and patients, respectively. Upregulated genes are coded in red, and downregulated genes are coded in green. RelB expression classification (low RelB vs high RelB) of each patient is represented on top of the heatmap. **E** RelB signature analysis using the Ingenuity Pathway Software (www.ingenuity.com) showing an enrichment in lipid and fatty acid (FA) metabolism linked genes. **F** Gene Ontology (GO) enrichment based on high RelB and low RelB expression as defined in (**A**).

To go one step further and generate a molecular portrait of RelB activation in HCC patients, we further explored whether gene expression profile (GEP) could subdivide these patients based on their RelB expression levels. Kaplan-Meier method was first used to define a subset of high-RelB HCC patients with a poor prognosis impact on overall survival (OS) and disease specific survival (DSS) in the TCGA cohort of HCC patients (TCGA-LIHC, *n* = 365) (Fig. 2A). There was no significant correlation between high-RelB expression and disease stages as evaluated by American Joint Committee on Cancer (AJCC 7th edition) (Fig. 2B). Interestingly, high RelB expression has also a strong prognosis impact at early stages of the disease (stage I and II) (Supplementary Fig. 2). Most importantly, the worse overall survival associated with the high-RelB HCC subset was validated in an independent cohort of patients with HCC (GSE 36376, *n* = 239) (Fig. 2C). In a second step, a hierarchical clustering algorithm was used to group genes based on high-RelB cases vs low-RelB cases. We identified 872 genes associated with RelB expression with a fold change ≥2 (Fig. 2D and Supplemental Table 2). Remarkably, a more detailed analysis of the high-RelB signature revealed increased expression of genes linked to lipid/FA metabolism (Fig. 2E, F), along with immunity, inflammation, and proliferation (Fig. 2E). These observations confirm that the differences in lipid metabolism signature identified in HCC cell lines translate to primary tumor biopsies.

### RelB is a sensor of lipid metabolism plasticity

**Fig. 3:**
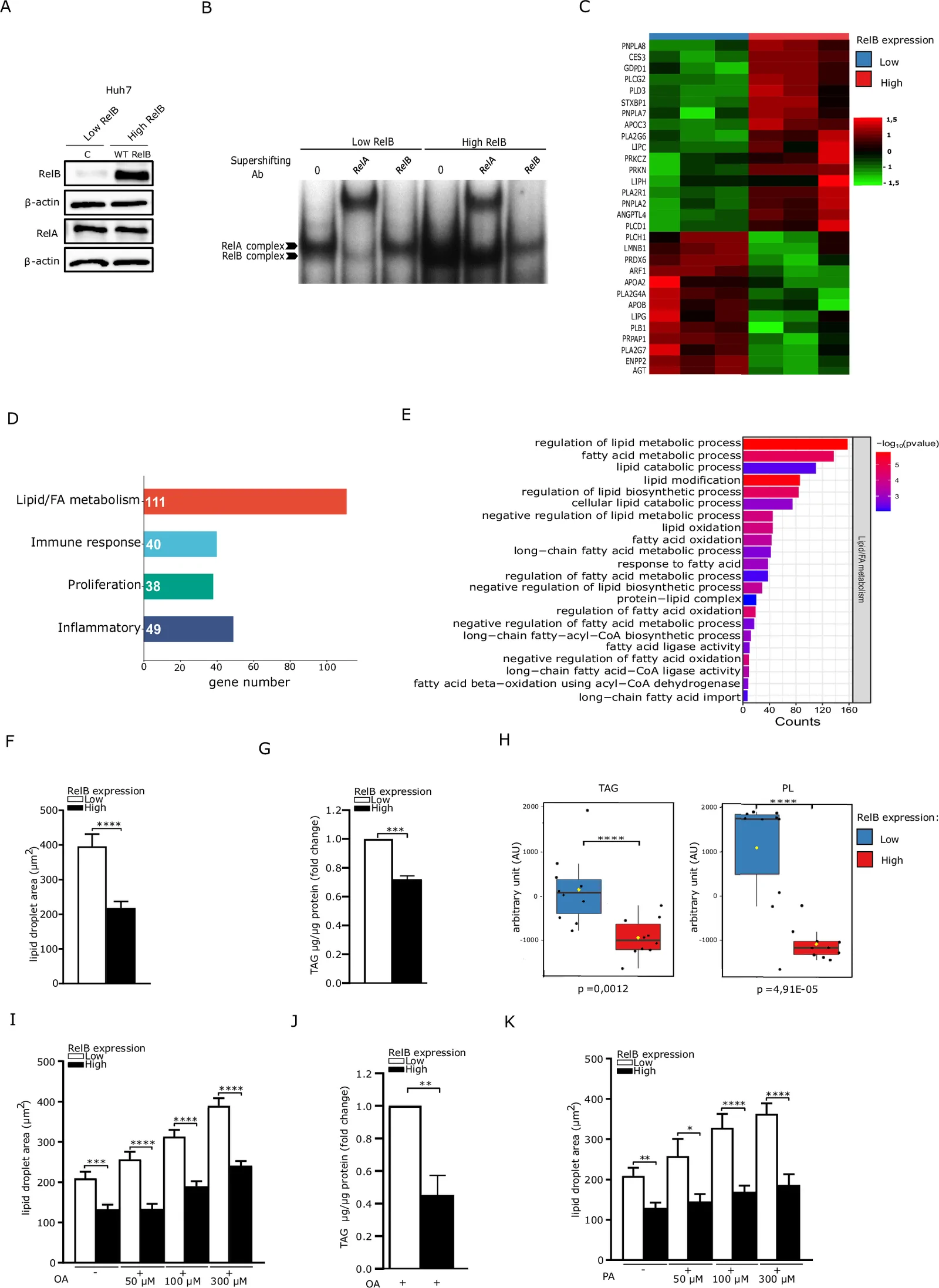

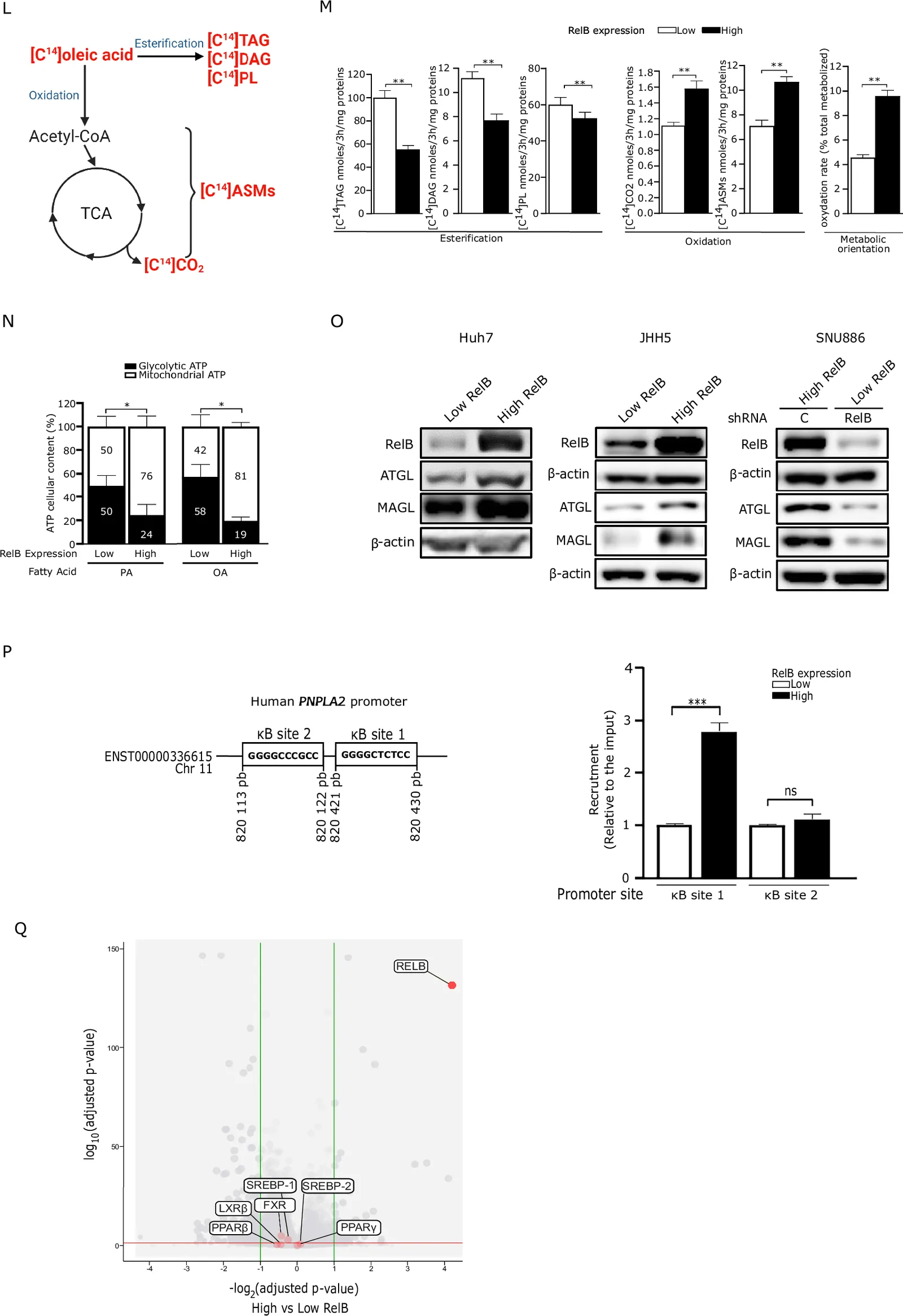
RelB is a sensor of lipid metabolism plasticity. **A** Huh7 cells stably transduced with lentiviruses encoding either RelB (WT RelB) or a control empty vector (**C**) was analyzed by immunoblotting for the indicated proteins. High-RelB indicates Huh7 cells expressing high level of ectopic RelB, low-RelB indicates Huh7 cells expressing low endogeneous levels of RelB (in accordance with the transcriptomic analysis of the CCLE dataset, see Fig. 1B). **B** Whole cell extracts from Huh7 cell lines, as described in (**A**), were analyzed for NF-κB binding activity using EMSA. For supershifts, extracts were incubated with the indicated antibodies before incubation with the labeled probe. RelB- and RelA-containing complexes are indicated. **C** High RelB expression defines a gene expression profile enriched in lipases and lipase-related genes. Upregulated genes are coded in red, and downregulated genes are coded in green. RelB expression classification (high RelB vs low RelB) of Huh7 cell lines is represented on top of the heatmap. Heatmap representing the relative expression of differentially expressed lipase and lipase-related genes (DEG) identified by RNAseq. Each column corresponds to an independent biological replicate (*n* = 3 per condition). Heatmap of DEGs generated from DESeq2 variance-stabilized counts after row-wise z-score scaling. **D** RelB signature analysis using the Ingenuity Pathway Software (www.ingenuity.com) showing an enrichment in lipid and fatty acid (FA) metabolism linked genes in high RelB Huh7 cells. Fold change ≥2. **E** Gene Ontology (GO) enrichment based on high RelB vs low RelB expression in Huh7 cells. **F** Lipid droplet content of the high-RelB and low-RelB Huh7 cells was quantified by measuring the area stained with Oil Red O. Magnification: x20. Error bars represent means ± SEM (*n* = 3). *P*-values by unpaired *t*-test, ****p < 0.0001. **G** Total TAG content of the high-RelB and low-RelB Huh7 cells was measured after lipid extraction by the Folch method. Fold change to relative level in low Huh7. Error bars represent means cells ± SEM (*n* = 3). *P*-values by Mann–Whitney tests, ***p < 0.001. **H** High-RelB and low-RelB Huh7 cells were monitored for intracellular triglycerides (TAG) and phospholipids (PL) using untargeted ^1^H NMR-based metabolomics. Boxplot of relative concentration, *n* = 10. *P*-values by unpaired *t*-test, ***p < 0.001, ****p < 0.0001. **I** Lipid droplet content in high-RelB and low-RelB Huh7 cells upon increasing concentrations of oleic acid (OA) Magnification: x20. Error bars represent means ± SEM (*n* = 3). *P*-values by unpaired *t*-test, ***p < 0.001, ****p < 0.0001. **J** Total TAG content of high-RelB and low-RelB Huh7 cells treated with exogenous oleic acid (50 μM) for 16 h was measured after lipid extraction by the Folch method. Fold change to relative level in low-RelB Huh7 cells. Error bars represent means ± SEM (*n* = 3). *P*-values by Mann–Whitney tests, **p < 0.01. **K** Lipid droplet content in high-RelB and low-RelB Huh7 cells upon increasing concentrations of palmitic acid (PA). Magnification: x20 (*n* = 3). Error bars represent means ± SEM (*n* = 3). *P*-values by unpaired *t*-test, *p < 0.05, **p < 0.01, ****p < 0.0001, ns: not significant. **L** Schematics depicting the fate of [^14^C]-oleic acid. Quantified metabolites derived from [^14^C]-oleic acid are marked in red. Esterification and oxidation processes are marked in deep blue. **M** Quantification of [^14^C]-oleic acid esterification rate into PL, DAG, and TAG (*left*), oxidation into CO_2_ and ASM (*center*), and metabolic orientation determined by the total oxidation rate on the total metabolized rate (*right*). TAG triglycerides, DAG diacylglycerol, PL phospholipids, ASM acid-soluble metabolites. Error bars represent means ± SD (*n* = 2 independent experiments performed in triplicate). *P*-values by Mann–Whitney tests, **p < 0.01. **N** High RelB expression rewires energetic metabolism towards mitochondrial ATP. ATP content of high-RelB and low-RelB Huh7 cells upon treatment for 16 h with exogenous oleic acid (OA, *n* = 2) or palmitic acid (PA, *n* = 3). Error bars represent means ± SD. *P*-values by unpaired *t*-test, *p < 0.05. **O** Whole cell extracts from high-RelB and low-RelB Huh7 (*left*), JHH5 (*center*), and SNU886 (*right*) cells were analyzed by immunoblotting for the indicated proteins; ATGL, *n* = 3, MAGL, *n* = 2. **P** High RelB expression promotes RelB recruitment to the *PNPLA2* promoter. Recruitment of RelB to the indicated κB sites in the *PNPLA2* promoter was examined by ChIP assays followed by qPCR analysis; *n* = 3, normalized to inputs. **Q** High RelB expression did not impact PPARs, LXRs, and SREBPs protein expression as monitored by quantitative mass-spectrometry-based proteomics in high vs low-RelB Huh7 cells, *n* = 5 per condition.

The up-regulation of selected genes encoding for lipases in high-RelB expressing HCC cells predicts potential differences in lipid metabolism compared to low-RelB expressing HCC cells. To search for disturbance of this metabolic program, we first generated a model in Huh7 cells (Huh7 belongs to the low-RelB cluster, see Fig. 1B) that mimics high RelB expression in HCC cells by stable lentiviral transduction (Fig. 3A and Supplemental Material). Importantly, Huh7 cells that ectopically express high RelB levels also resulted in a marked induction in RelB activity without affecting expression (Fig. 3A) and activity (Fig. 3B) of the canonical RelA NF-κB subunit. To further characterize this cellular model, we performed a RNAseq analysis and showed that increased RelB activity in Huh7 cells induced a strong enrichment of the transcription of genes linked to lipase (Fig. 3C and Supplemental Table 3), just as what was seen in the large panel of HCC cell lines (Fig. 1), indicating that our high-RelB Huh7 cell model reflects RelB functions in naturally occurring high-RelB HCC cells. In addition, we identified an increased expression of genes linked to lipid/FA metabolism (Fig. 3D, E), along with immune response, inflammation and proliferation. Notably, the GO terms on genes linked to lipid/FA metabolism included regulation of lipid metabolic process, FA metabolic process, lipid catabolic process and regulation of FAO (Fig. 3E), just as what was seen in the subset of high-RelB HCC patients (Fig. 2E). These data suggested that RelB could rewire the lipid metabolism from lipid storage to FAO via the control of lipase expression. To test this hypothesis, we first assessed the impact of RelB activation on lipid droplet content, the intracellular reservoir of lipids [15]. Remarkably, high RelB expression induced a marked decrease in lipid droplets (Fig. 3F). Similarly, a decrease in intracellular TAG was also observed upon increased RelB expression in Huh7 cells (Fig. 3G). In addition, using an untargeted NMR-based metabolomics approach, we monitored the metabolic changes occurring upon RelB activation in Huh7 cells. Ectopic expression of RelB led to a massive depletion of intracellular TAG and phospholipids (PL) (Fig. 3H). The RelB-dependent functional differences in neutral lipid storage predict differences in FA handling. We first evaluated the impact of RelB on lipid storage upon increasing amount of FA. In high-RelB Huh7 cells, lipid storage was significantly decreased in response to exogenously supplied monounsaturated FA (Oleic acid, Fig. 3I, J) and saturated FA (Palmitic acid, Fig. 3K) compared to what was seen in RelB-low expressing cells.

To reinforce our observations on the impact of RelB on lipid storage in Huh7 cells, we have generated a second model of high RelB expression in JHH5, a cell line that also belongs to the low-RelB cluster (see Fig. 1B) by stable lentiviral transduction (Supplementary Fig. 3A, B, and Supplemental Material). High ReB expression in JHH5 cells resulted in a marked induction in RelB activity without affecting the canonical NF-κB/RelA expression and activity (Supplementary Fig. 3A, B). Most importantly, just as was seen in Huh7, high RelB expression in JHH5 cells induced a marked decrease in lipid droplets and intracellular TAG (Supplementary Fig. 3C, D). We next developed a stable RelB knockdown approach in SNU886 cells (SNU886 belongs to the high-RelB cluster, see Fig. 1B) using either a lentivirus carrying a shRNA targeting RelB or a shRNA control (Supplementary Fig. 3E, F). Of note, RelB expression knockdown in SNU886 cells resulted in a marked reduction in RelB DNA binding activity without affecting the expression and activity of the canonical NF-κB RelA subunit (Supplementary Fig. 3E, F). Most importantly, RelB knockdown resulted in a marked and significant increase in lipid storage as measured by lipid droplets (Supplementary Fig. 3G) and intracellular TAG levels (Supplementary Fig. 3H) upon supplying oleic acid.

To provide independent and parallel evidence for a differential utilization of FA upon high RelB expression, a targeted ^14^C-oleic acid approach was undertaken, and ^14^C enrichment was assessed in a defined set of metabolites derived from FA metabolism (Fig. 3L). Very interestingly, the relative levels of ^14^C enrichment for TAG and DAG were significantly decreased upon high RelB expression, whereas acid-soluble metabolites (ASM) and CO_2_ levels were strongly increased (Fig. 3M). This is consistent with a greater entry of FA into FAO to promote mitochondrial activity, at the expense of esterification, the reaction that converts FA into neutral lipids, such as TAG, for lipid storage in lipid droplets. Altogether, these observations demonstrate the central role played by RelB into the metabolic reprogramming in HCC cells from lipid storage to FAO. Moreover, to better understand the energetic consequences induced by high RelB activation, we evaluated how RelB impacts on the level of production of glycolytic vs mitochondrial ATP in HCC cells. Compared to low-RelB expressing Huh7 cells, high-RelB expressing cells derive a significantly higher portion of their total energy from mitochondrial oxidative metabolism than glycolysis (Fig. 3N). Altogether, these data indicate that RelB exerts a critical role in the energetic reprogramming of HCC cells and is a sensor of lipid metabolism.

To further assess the underlying mechanisms, it was important to determine how RelB expression impacted on the protein expression levels of central lipolytic lipases [16], namely ATGL and MAGL, in all three HCC cell models that we have generated. Remarkably, increased RelB expression in Huh7 and JHH5 cells increased ATGL and MAGL protein expression levels (Fig. 3O, *left* and *central*, and Supplemental material). Vice versa, RelB expression knockdown in SNU886 cells led to a marked decrease in ATGL and MAGL protein expression (Fig. 3O*, right*, and Supplemental material). Further evidence for a direct role for RelB in regulating *PNPLA2* transcription was obtained by chromatin IP (ChIP) analysis. As shown in Fig. 3P, RelB is efficiently recruited to the *PNPLA2* promoter upon high RelB expression. Last, since PPARs, LXRs, and SREBPs are important regulators of lipid metabolism, we evaluated by quantitative proteomics whether RelB might impacts on the protein expression levels of these factors. Importantly, high RelB expression had no significative impact on either PPARs, or LXRs, or SREBPs protein levels (Fig. 3Q), strongly suggesting that the novel role for RelB in the rewiring of lipid metabolism that we have highlighted is not directly linked to an impact of these other metabolic regulators.

Taken together, these results strongly suggest that RelB promotes lipid unstorage from lipid droplets and induction of FAO through, at least in part, the direct control of lipolytic lipase expression.

### RelB-dependent reprogramming of energy homeostasis in HCC cells impacts on tumor development

**Fig. 4:**
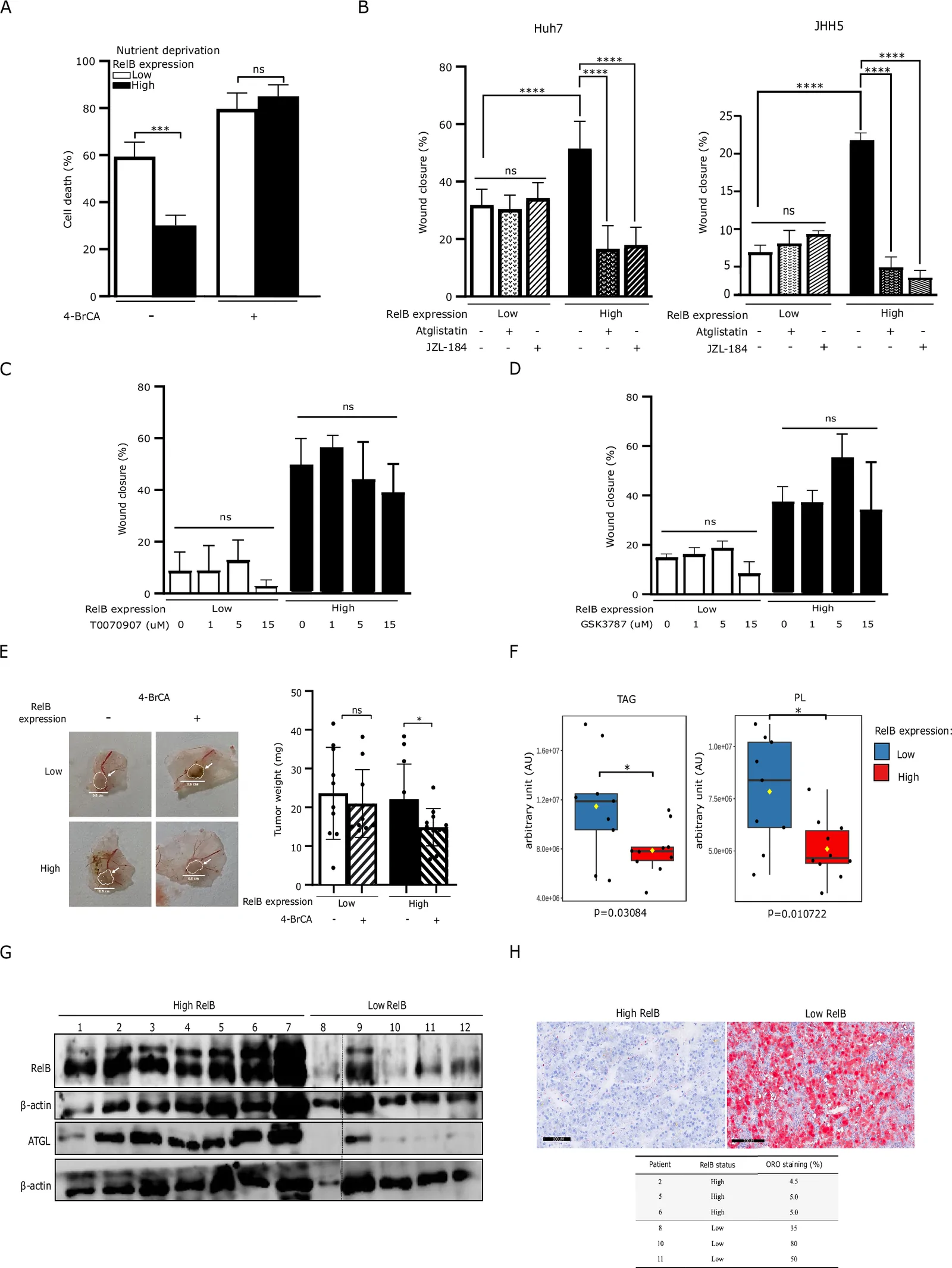
RelB-dependent reprogramming of energy homeostasis towards FAO in HCC cells impacts on tumor development. **A** High-RelB and low-RelB Huh7 cells were grown for 48 h upon FBS deprivation with or without 4-BrCA (10 μM). Cell death was assessed upon DAPI staining. Error bars represent means ± SEM (*n* = 3). *P*-values by unpaired *t*-test, ***p < 0.001, ns not significant. **B** High-RelB and low-RelB of either Huh7 (*left*) or JHH5 (*right*) cells treated with exogenous oleic acid as described in Methods, and the indicated inhibitors or left untreated, were analyzed for cell migration using scratch-wound assays. Wound closure was measured 48 h post-scratch, and the percentage of wound closure is presented. Error bars represent means ± SD (*n* = 3). *P*-values by unpaired *t*-test, ****p < 0.0001, ns not significant. High-RelB and low-RelB Huh7 cells treated with exogenous oleic acid as in (**B**), and increasing amounts of either an irreversible PPARγ (**C**) or PPARβ/δ (**D**) inhibitor, or left untreated were analyzed for cell migration using scratch-wound assays. Wound closure was measured 48 h post-scratch, and the percentage of wound closure is presented. Error bars represent means **±** SD (*n* = 3). *P*-values by unpaired *t*-test, ns not significant. **E** High RelB expression renders tumors highly susceptible to FAO inhibition. Tumor development in vivo using the CAM model. High-RelB and low-RelB Huh7 cells were engrafted on the CAM and treated by 4-BrCA (10 µM) at days 3 and 5 post-engraftment, *n* = 8–12 tumors, 2 independent experiments. Representative tumor images at day 16 are shown (*left*). Tumor weight of individual tumors is represented for the indicated conditions (*right*). Error bars represent means **±** SD (*n* = 2). *P*-values by Mann–Whitney tests, *p < 0.05, ns not significant. **F** High-RelB and low-RelB Huh7 tumors isolated from the CAM model in (**E**) were monitored for intracellular triglycerides (TAG) and phospholipids (PL) using untargeted ^1^H NMR-based metabolomics. Boxplot of relative concentration, *n* = 10. *P*-values by unpaired *t*-test, *p < 0.05. **G** High RelB expression is associated with increased ATGL protein expression in HCC patients. Whole cell extracts from HCC patient samples were analyzed by immunoblotting for the indicated proteins, *n* = 12 HCC patients. **H** High RelB expression is associated with decreased lipid droplet content in HCC patient samples. Oil Red O staining on high-RelB and low-RelB HCC patient samples, frozen sections. Cut off ≥4.5%. A representative image is shown. Scale bar is indicated.

In a next step, we determined whether the RelB-dependent energy switch represented a means for HCC cells to increase their survival upon metabolic stress. Nutrient deprivation-induced cell death was markedly decreased upon high RelB expression in Huh7 cells (Fig. 4A). Remarkably, the RelB-dependent prosurvival function is abolished upon treatment with 4-BrCA, an inhibitor that irreversibly inhibits oxidation of long-, medium-, and short-chain FA [17] (Fig. 4A), indicating that RelB promotes the ability of HCC cells to meet their metabolic demand during nutrient starvation via FAO, thus avoiding a metabolic crisis and cell death.

Next, we explored the functional relevance of RelB on cell migration, which is important during the whole cascade of cancer development. A significant increase in wound closure was observed upon high RelB expression in Huh7 and JHH5 cells as compared to what was seen in the low-RelB expressing cells (Fig. 4B). Further, since we observed that high RelB expression promotes transcription and protein expression of ATGL and MAGL (Fig. 1D, F and Fig. 3O, P, and Supplemental Material), the two major lipases controlling lipolysis [16] (see scheme Fig. 1E), we hypothesized that the pro-migration function of RelB could rely on ATGL and MAGL activity [18]. Remarkably, treatment with ATGL or MAGL inhibitors (ATGLstatin and JZL184, respectively) strikingly decreased the migration of both high-RelB expressing Huh7 and JHH5 cells (Fig. 4B) with no effect on low-RelB expressing cells. To rule out an involvement for PPAR activity in the RelB-dependent HCC cell migration, Huh7 cells were first treated with increasing amounts of the irreversible PPARγ antagonist T0070907 (Fig. 4C). Inhibition of PPARγ has not significant effect on RelB-dependent increase in Huh7 cell migration. Similar observations were made upon inhibition of PPARβ/δ with GSK3787 treatment (Fig. 4D). Taken together, these results strongly suggest that high RelB expression promotes HCC cells' migration via the control of expression and activity of central lipases (namely ATGL and MAGL), thereby leading to a profound rewiring of lipid metabolism from storage to usage as a major source of FA to feed FAO. To directly demonstrate that RelB-dependent FAO impacts on tumor growth, we took advantage of the well-recognized in vivo chorioallantoic membrane (CAM) model [19]. Although having little effect on basal tumor growth, high RelB activation renders tumors highly susceptible to FAO inhibition (Fig. 4E). In addition, using an untargeted NMR-based metabolomics approach, we monitored the metabolic changes occurring upon RelB activation in CAM tumors. High RelB expressing tumors exhibited a significant depletion of intracellular TAG and phospholipids (PL) as compared to low expressing tumors (Fig. 4F). Collectively, these findings indicate that boosting FA metabolism in established cancer cells through increased RelB expression promotes tumorigenesis in vivo. Ultimately, a significant accumulation of ATGL protein was observed in a cohort of HCC patients expressing high levels of RelB as compared to low expressing patients (Fig. 4G and Supplemental Material). In line, a marked decrease in lipid droplet content was observed in high-RelB expressing HCC patient samples as compared to low RelB patient samples (Fig. 4H). These observations indicate that the differences in lipid metabolism identified in HCC cell lines translate to primary tumor biopsies.

**Fig. 5:**
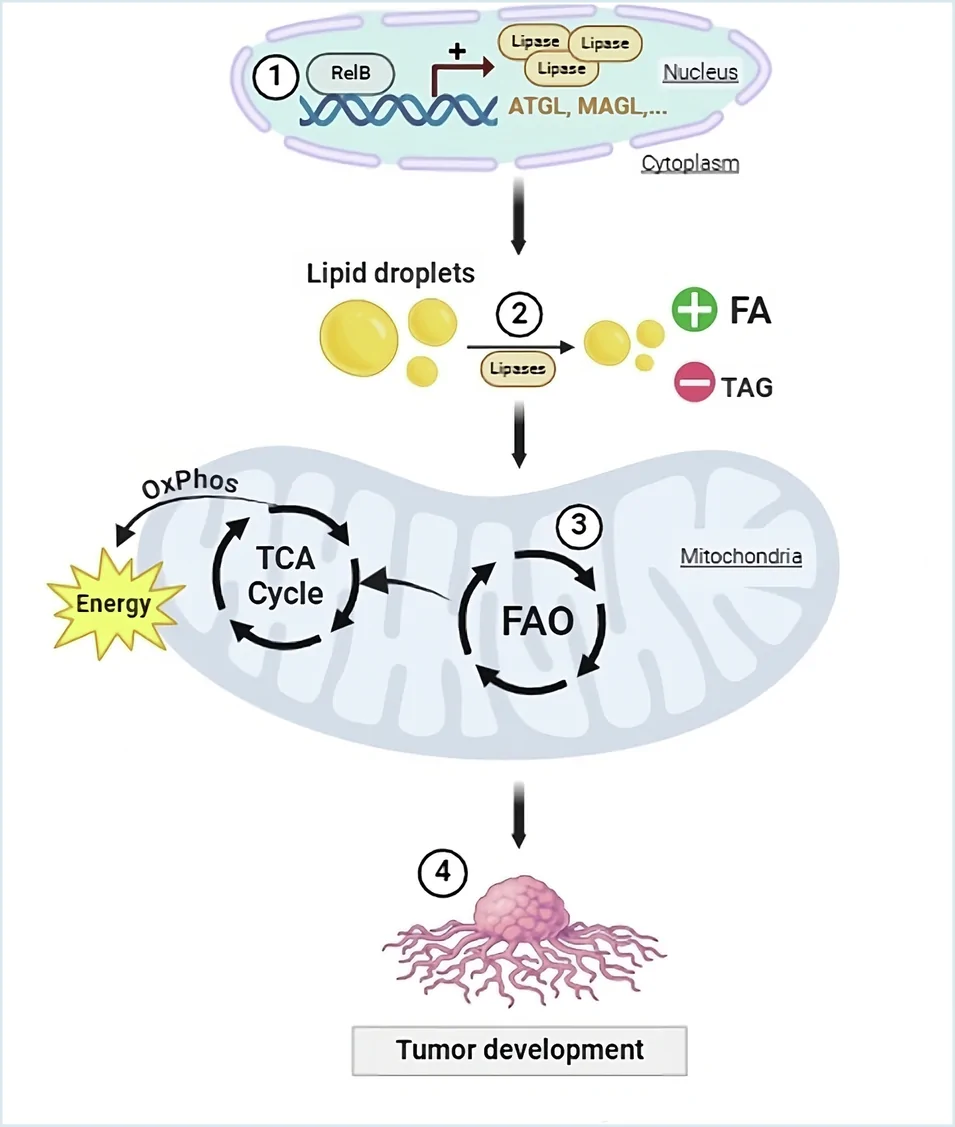
Schematic representation of the impact of RelB on lipid metabolism plasticity, and energy reprogramming. We have uncovered that RelB is a central sensor of lipid metabolism plasticity in HCC cancer cells. RelB controls the expression of central lipases (1), leading to increased lipolysis (2), thereby driving cancer cells to oxidize fatty acids (FAO) for mitochondrial activity and energy production (3). The RelB-dependent rewiring of lipid metabolism towards FAO promotes tumor development (4). ATGL adipose triglyceride lipase, MAGL monoglyceride lipase, FA fatty acid, TAG triglycerides, FAO fatty acid oxidation, TCA tricarboxylic acid cycle, OxPhos oxidative phosphorylation.

As summarized in Fig. 5, we have uncovered that RelB is a central sensor of lipid metabolism plasticity in HCC cancer cells. RelB drives transcription of central lipases (1), leading to increased lipolysis (2), thereby driving cancer cells to increased FAO for mitochondrial activity and energy production (3). The RelB-dependent rewiring of lipid metabolism towards FAO promotes tumor development (4) [4].

## Discussion

Reprogramming of cancer cell metabolism is now a recognized hallmark of cancer. Recently, lipid metabolism has emerged as a central player in altered energetics in cancers affecting cancer cell survival and tumor development [3, 4, 20]. Our study reveals that the alternative RelB NF-κB subunit plays a central role in the control of lipid metabolism plasticity at the crossroads of lipid storage and its use as a major source of FA to feed the process of FAO in the mitochondria. To our knowledge, this is the first report of a role for RelB in lipid metabolism reprogramming in cancer cells.

Few studies have reported the ability of the classical NF-κB pathway to promote either mitochondrial respiration [21–24] or Warburg effect [25] in cancer cells. However, an involvement of classical NF-κB in the control of lipid metabolism is currently unknown. In contrast, a role for the alternative NF-κB pathway in cancer cell metabolism remains almost undefined. The nuclear factor-κB-inducing kinase (NIK) is an upstream critical regulator of the alternative NF-κB pathway that leads mainly to RelB activation [7, 26]. Although NIK was identified as a critical regulator of mitochondrial fission and metabolic adaptation of cancer cells [27–29], these effects were unexpectedly independent on NF-κB. We have been the first to report that RelB constitutive activation can reprogram diffuse large B-cell lymphoma (DLBCL) metabolism towards oxidative energy metabolism in DLBCL cells and protects DLBCL cells from antimetabolic drugs-induced apoptosis [30]. A recent study suggested that RelB contributes to tamoxifen resistance in breast cancer cells via the upregulation of GPX4 [31]. A preprint indicated that RelB overexpression in HepG2 cells under specific metabolic stress induced by chronic lipid-rich culture conditions induces a significant decrease in lipid accumulation [32]. However, how RelB impacts on lipid metabolism in cancer cells still remained a central unanswered question.

Our study reveals that RelB plays a central role at the crossroads of lipid storage and release of FA from the lipid droplets to feed FAO and mitochondrial energetic metabolism. First, we have shown that RelB expression correlates with the transcription of several lipases and lipase-related genes, and high RelB activity is linked to increased protein expression levels of ATGL and MAGL, the two central lipases involved in lipid droplet breakdown via lipolysis. Second, high RelB activation strongly decreases lipid droplet and TAG content. Third, high RelB activity rewires HCC cell metabolism to FAO at the expense of FA esterification and provides an energetic source to feed the mitochondrial respiration. Fourth, ATG and MAGL activities are required for increased HCC cell migration upon high RelB activation. Fifth, RelB-dependent increase in FAO protects HCC cells against nutrient deprivation-induced cell death and provides an essential signal for tumor growth. Sixth, high-RelB protein expression levels correlate with high ATGL expression and decreased lipid storage in HCC patients.

It is not clear yet how RelB expression levels are controlled in HCC cells. Post-translational modifications of RelB, such as phosphorylation, SUMOylation, and polyubiquitination, were reported to control RelB stability and function [26, 33]. Additional factors most probably control overall RelB protein expression levels, such as the proteosomal degradation of its main inhibitor p100 [34–36], RelB cleavage by endopeptidases and caspases [37, 38], and chromatin remodeling [39].

Beyond HCC, abnormal RelB activity has been reported in several solid cancers, including glioma, breast, prostate, and colon cancer [40–45] as well as in hematopoietic malignancies such as multiple myeloma and DLBCL [46, 47]. Further abnormal RelB expression has been linked to increased several cancer aggressivity and poor patient outcome [40, 42, 44, 45, 47–52]. Thus, it is likely that the RelB-dependent lipid/FA dynamics and associated pro-survival and tumorigenic effects may be generalized to other neoplasms, especially those addicted to alternative NF-κB.

In summary, we established that RelB is a central player in lipid metabolism plasticity and energy homeostasis in HCC cells. Our data are of great functional importance because they constitute a significant advance in the understanding of RelB function in cancer, and provide a strong rationale for the development of new molecules targeting RelB for therapeutic intervention in cancer.

## Methods

### Reagents

Oleic acid (01008), palmitic acid (P0500), L-carnitine hydrochloride (C0283), 2-deoxy-D-glucose [2-DG] (D8375), Oil Red O (O0625), and mitomycin C (M4287) were purchased from Merck/Sigma Aldrich. 4’,6-diaminidino-2-phenylindole [DAPI] from Molecular Probes. 4-Bromobut-2-enoic acid [4-BrCa] (A698521) was purchased from Ambeed; Atglsitatin (HY-15859) and JZL184 (HY-15249), T0070907 (HY-13202), and GSK3787 (HY-15577) from MedChemExpress.

### Antibodies

Antibodies are annotated as follows: WB, western blot; ES: EMSA/supershift, ChIP: Chromatin IP. Dilutions are indicated. The following primary antibodies were used: RelB (Santa Cruz Biotechnology, sc-226, clone C-19, WB 1/1000); RelB (Santa Cruz Biotechnology, sc-226 X, clone C-19, ES 1/8,5); RelB (Cell Signaling Technology, 10544, clone D7D7W clone, ChIP 10 μl per 10 μg of chromatin); RelA (Santa Cruz Biotechnology, sc-372, clone C-20, WB 1/1000); RelA (Santa Cruz Biotechnology, sc-8008 X, clone F-6, ES 1/8,5); ATGL (Santa Cruz Biotechnology, sc-365278, clone F-7, WB 1/1000); MGL (Santa Cruz Biotechnology, sc-398942, clone C-11, WB 1/1000); and β-actin (Santa Cruz Biotechnology, sc-47778, clone C-4, WB 1/1000).

### Plasmid constructs

Expression vector for human RelB was obtained from M. Körner (Institut André Lwoff, Villejuif, France), and pSuper vector containing the polymerase III H1promoter was provided by T. Tuschl (The Rockefeller University, New York). pTRIP–RelB was generated by subcloning human full-length (FL) RelB cDNA into pTRIP–ΔU3–EF1α–IRES GFP lentiviral vector [33] following standard recombinant DNA procedures. pTrip-shRNA RelB was generated by subcloning an oligonucleotide designed to target RelB under control of the H1 promoter into pTRIP-ΔU3-MND-GFP lentiviral vector [53]. pTRIP–shRNA control was generated following the same approach using a scrambled control oligonucleotide. RelB sense 5′- GATCCCCGGAAATCATCGACGAATACTTCAAGAGAGTATTCGTCGATGATTTCCTTTTTGAATTCA-3′; RelB antisense 5′-AGCTTGAATTCAAAAAGGAAATCATCGACGAATACTCTCTTGAAGTATTCGTCGATGATTTCCGGG-3′; control sense 5′-GATCCCCCGTACGCGGAATACTTCGATTCAAGAGATCGAAGTATTCCGCGTACGTTTTTGAATTCA-3′; control antisense 5′-AGCTTGAATTCAAAAACGTACGCGGAATACTTCGATCTCTTGAATCGAAGTATTCCGCGTACGGGG-3′.

### Cell lines and culture conditions

Huh7, JHH5, and SNU886 cell lines validated using whole exome sequencing and tested mycoplasmas free were provided by J. Zucman-Rossi (Liver Cancer Cell Lines Database, https://lccl.zucmanlab.com/hcc/home) [54]. Cells were grown in MEM culture medium (41090-028 Gibco) supplemented with 10% of fetal bovine serum [FBS], 100 U/mL penicillin, and 100 mg/mL streptomycin (15140-122 Gibco), 25 mM Hepes Buffer (15630-056 Gibco), 1 mM of sodium pyruvate (ref 11360-039 Gibco), 1% (v/v) of non-essential amino-acids [NEAA] (ref 11140-050 Gibco), which stands for standard culture medium. For exogenous fatty acid treatment, exponentially growing Huh7 cells were serum-starved for 5 h, and then oleic or palmitic acid with 1% (w/v) of fatty acid-free bovine serum albumin (BSA) (A7030 Sigma Aldrich) was added for 16 h.

### Immunoblotting

Cells were collected in whole cell lysis buffer (400 mM NaCl, 25 mM Hepes pH 7.7, 1.5 mM MgCl_2_, 0.2 mM EDTA pH 8, 1% NP-40, 2 mM DTT, 20 mM β-glycerol phosphate, 100 μM Na_3_VO_4_, 10 mM, PNPP 1 mM PMSF, 1X cOmplete protease inhibitor cocktail (Roche Diagnostic). Cell lysates (20 μg of protein per lane in 2X loading buffer containing bromophenol blue) were resolved on 7.5%–10% SDS–polyacrylamide gels. On each gel, a size marker was run: 3 µl PageRuler (Thermo Fisher Scientific, 26616). Proteins were then transferred to nitrocellulose membrane (Amersham Protran 0.45 μm) by liquid transfer using 1X transfer buffer (25 mM Tris, 195 mM Glycine, 10% ethanol v/v in H_2_O). Membranes were blocked with 5% non-fat skimmed milk powder (Régilait) in 0.1% Tween-20 and 1× PBS for 1 h at room temperature. Membranes were cut at the appropriate marker size to enable the probing of several antibodies on the same membrane. Blots were then probed with the relevant primary antibodies in 5% BSA, 0.1% Tween-20, and 1× PBS at 4 °C overnight with gentle motion. Membranes were washed with 0.1% Tween-20 and 1× PBS 3 times 5 min at room temperature, and incubated with horseradish-peroxidase-conjugated secondary antibodies (Bio-Rad) in 5% BSA, 0.1% Tween-20, and 1× PBS for 1 h at room temperature, and washed 3 times 5 min with 0.1% Tween-20 and 1× PBS. Antigens were detected using ECL (Pierce, 32106), ECL plus (Pierce, 32132), and ECL SELECT (Cytiva, 12173679774) chemiluminescent detection kits. Signals were recorded using an Amersham Imager 600. Images were processed using Fiji 2.0.0-rc-69/1.52n software. Full scans of blots are provided in the Supplemental Material.

### Lentiviral production and transduction

Production of infectious recombinant lentiviruses was performed by transient transfection of 293 T cells as previously described [55]. For infection, 400,000 cells per 35 mm plate were incubated overnight with recombinant lentiviruses. An equal amount of fresh culture medium was added 24 h later, and after 48 h, cells were washed and seeded in fresh culture medium. GFP positive cells were sorted with FACSAria™ sorter (Becton Dickinson).

### Electrophoretic Mobility Shift Assays (EMSA) for NF-κB

Protein cell extracts were analyzed for DNA binding activity using a 31-mer κB oligonucleotide which contains the two direct repeats of the HIV enhancer sequence from the position −107 to −80 (5’-ACAAGGGACTTTCCGCTGGGGACTTTCCAGG-3’) as κB probe [36]. The oligonucleotide probes were end-labelled using ATP-γP^32^ with polynucleotide T4 kinase (Roche, 709557). In a typical experiment, 10–15 μg of protein extracts were incubated with the ^32^P-end labelled probe for 40 min in 10 mM Tris-HCl, pH 7.6, 100 mM NaCl, 1 mM EDTA, 5 mM DTT, 2% glycerol, 0.2 μg/ml poly dI:dC. The bound oligonucleotide was separated from the free probe by electrophoresis on polyacrylamide gel. For supershift assays, protein extracts were incubated with specific antibodies for 30 min on ice before incubation with the labeled probe.

### ChIP-seq Atlas data analysis

RelB recruitment to the promoter regions of genes encoding for lipases was examined using ChIP-seq Atlas database and available data from GSE 55105 [56]. Visualization using IGV browser.

### Chromatin immunoprecipitation (ChIP) assays

Cells were harvested, washed once in PBS and spined down at 2000 rpm for 5 min at 4 °C. The pelleted cells were resuspended in buffer 1 (50 mM Tris pH 8.0, 1 mM EDTA, 0.4% NP40, 10% glycerol, cOmplete 1X, 0.5 mM DTT, 1 mM PMSF), incubated for 15 min at 4 °C and centrifuged at 3000 rpm for 5 min at 4 °C for nuclei isolation. The pelleted nuclei were crosslinked with 1% formaldehyde (Sigma F8775) in 1X cold PBS for 10 min at 4 °C on a rotating wheel. Then, 2.5 M glycine (Sigma, 50046) was added to a final concentration of 0.125 M and incubated at 4 °C for 5 min on a rotating wheel followed by centrifugation at 3000 rpm for 5 min at 4 °C. After two washes in 1X cold PBS, 1 mM PMSF, pellets were collected by centrifugation at 1500 × *g* for 5 min at 4 °C. Pellets were then sheared in buffer L2 (50 mM Tris-HCl pH 8.0, 5 mM EDTA pH 8.0, 1% SDS, cOmplete, 1 mM PMSF) to approximately 200–600 bp average size using a Bioruptor Pico (Diagenode). After centrifugation at 13,000 rpm at 4 °C for 2 min, supernatants containing sheared chromatin were used for immunoprecipitation. Twenty-five μl (10%) of sheared chromatin was used as input DNA to normalize sequencing data. Chromatin immunoprecipitation (at least 50 μg of chromatin per ChIP condition) was carried out using sheared chromatin and the anti-RelB antibody in buffer DB (50 mM Tris-HCl pH 8.0, 5 mM EDTA pH 8.0, 200 mM NaCl, 0.5% NP40, cOmplete, 1 mM PMSF) overnight at 4 °C, which was subsequently complexed with protein A sepharose beads (Cytiva 17-0780-01) saturated with salmon sperm DNA for 1 h. After spinning and removal of supernatants, beads were successively washed in buffer WB (20 mM Tris-HCl pH 8.0, 2 mM EDTA pH 8.0, 0.1% SDS, 1% NP40, 500 mM NaCl, four times), and TE (10 mM Tris-HCl pH 8.0, 1 mM EDTA pH 8.0, three times). Finally input and immunoprecipitated chromatin were resuspended in buffer EB (1X TE, 2% SDS), de-crosslinked by heating at 65 °C overnight and subjected to proteinase K (Invitrogen, 25530-049) treatment. Once salmon sperm DNA was added to the de-crosslinked immunoprecipitated chromatin, DNA isolation was performed using the QIAquick PCR purification kit (Qiagen, 28104). Samples were analyzed by quantitative PCR. About one-fortieth of the immunoprecipitated DNA was used in each PCR.

To search for candidate κB sites in the promoter of human *PNPLA2* gene, we performed an in silico motif scan using the JASPAR 2024 Core Database using the RelA matrix MA0107.1 and the RelB matrix MA1117.1 with a relative profile score threshold of 80–90%. The in silico predictions revealed 2 main κB sites with high-confidence matches, the GGGGCTCTCC κB site 1 at positions 820421-820430 (*p*-value: 0.000012, *q*-value: 0.00525), and the GGGGCCCGCC κB site 2 at positions 820113-820122 (*p*-value: 0.0000759, *q*-value: 0.00862). These candidate NF-κB binding sites were used for experimental validation by ChIP using the following promoter-specific primers: PNPLA2 κb site 1 sense 5′-CAAGGGCCCGGAGGAG-3′; PNPLA2 κb site 1 antisense 5′-CAAGGAGAGGGCAGGAGAG-3′; PNPLA2 κb site 2 sense 5′-TTTGTTTTGCTCCGAGGGTG-3′; PNPLA2 κb site 2 antisense 5′- ATTAAGCAGCTTTTGCTGCAC-3′. Quantitative PCR was performed using the FastStart DNA SYBR Green Master (Roche, ref. 03515885001) using 40 ng of each primer in a final volume of 25 μl according to the manufacturer’s instructions. The PCR conditions were as follows: 9 5 °C 10 min, followed by 35–38 cycles at 95 °C 10 s, 60 °C 5 s and 72 °C 10 s, and final elongation at 72 °C 10 min.

### Lipid droplet content

Fixed cells with formalin 10% (v/v) and isopropanol 60% (v/v) were stained with Oil Red O for 10 min at room temperature. Image acquisition was performed using optical microscopy (magnification: x20), and quantification of Oil Red O staining was made using image J software (V1.53c) and expressed as lipid droplet area.

### Total triglyceride content

Lipids were extracted using the Folch method using chloroform/methanol (2:1, v/v). Triglyceride content was quantified using a colorimetric diagnostic triglyceride assay kit (Triglycerides FS; Diasys) and further normalized to whole cell extract protein concentration as measured by BCA Protein Assay kit (23227, Pierce).

### Fatty acid metabolic flux

[1-^14^C]Oleic acid was obtained from GE Healthcare. Huh7 cells were treated for 16 h with exogenous oleate (50 µM), and then oleate oxidation and esterification rates were determined during the last 3 h of culture in the presence of 300 µM of [1-^14^C] oleate (0.5 Ci/mol) bound to 1% (w/v) BSA and 1 mM carnitine. At the end of the 3 h incubation period, the medium was collected to determine [^14^C]CO_2_ and [^14^C]acid-soluble metabolites (ASMs) by scintillation counting [57] (Packard). ASMs essentially consist of acyl-carnitines, Krebs cycle intermediates, and acetyl-CoA. Oleate oxidation rates were calculated from [^14^C]CO_2_ and [^14^C]ASMs and expressed as nmoles of oleate oxidized over 3 h per mg of protein. For esterification rates, cells were washed and scraped in PBS to determine [^14^C]TAG, [^14^C]phospholipids (PL) and [^14^C]diacylglycerols (DAG). Lipids were extracted with chloroform/methanol (2:1, v/v) and separated by thin-layer chromatography on silica-gels plates [57] (Merck). TAG, DAG and PL were identified using standard oils and coloration with iodine vapour. Specific bands for each fraction were cut and placed in scintillation liquid, and ^14^C-labelled PL, DAG and TAG products were quantified by radioactivity counting. Protein content was determined using the BCA protein assay (Thermo Fisher Scientific).

### Measurement of intracellular ATP content

Intracellular ATP content was measured using an ATP luminescence detection assay kit (CellTiter-Glo^®^ Luminescent Cell Viability Assay, Promega, Madison, WI, USA) according to the manufacturer’s instructions. Briefly, around 4 × 10^3^ cells were seeded (96 well plates) in 100 µL of cell culture medium (DMEM with no glucose, ref A1443001 supplemented with 10% FBS, glucose (1 g/L), L-carnitine (1 mM), 100 U/mL penicillin, 100 mg/mL streptomycin, 25 mM Hepes Buffer) with or without exogenous fatty acid and 1% (w/v) fatty acid-free BSA for 16 h. Cells were treated for 1 h 30 min with or without 20 mM 2-deoxy-D-glucose (2-DG) at 37 °C followed by 30 min at room temperature to inhibit glycolysis. At the end of the incubation time, 100 µL of CellTiter-Glo solution were added, and cells were incubated for 10 min at room temperature. The luminescence signal (relative luminescence units (RLU)) was recorded with a microplate reader (Centro LB 960 microplate luminometer, Berthold Technologies, Bad Wildbach, Germany). The difference between total ATP content and ATP produced under 2-DG treatment results in glycolytic ATP.

### Scratch-wound assays

Cells were grown to a confluent monolayer for 24 h in complete MEM medium with 10% (v/v) FBS, and then cells were grown in 0% FBS for an additional 5 h before scratches were made using a 0.5 mm-diameter pipette tip to slow down proliferation. Two hours before the scratches, mitomycin C (14.9 nM) was added with or without Atglistatin (40 µM) or JZL184 (0.5 µM). After the scratch, the medium was replaced with MEM medium containing no FBS with or without Atglistatin or JZL184 at the same concentration. Scratch area was monitored over 48 h. Scratch assays were quantified using the MRI wound healing tool of ImageJ (V1.54i).

### Cell viability assay

Cell viability was assessed using 4’, 6-diamidino-2-phenylindole (DAPI) 1 µg/mL staining for 5 min at room temperature with stained (non-viable) and unstained (viable) cells.

### CAM model experiment

Fertilized chicken eggs (*Gallus domesticus*) were incubated at 37.7 °C and 55% humidity, laid vertically and turned 180° every hour for 8 days. On day 8 of embryonic development, an about 1 cm^2^ window on the eggshell was performed, careful not to disrupt the remaining structure, and then covered with tape for further incubation. High-RelB and low-RelB Huh7 cells growth in condition of exogeneous FA (see above, cell culture conditions) were harvested, resuspended in MEM/Matrigel 50/50 vol/vol (25 μL), and engrafted on the CAM (*n* = 10 to 12 eggs/condition). CAM presenting a tumor at day 12 were treated with or without 4-BrCA at days 12 and 14. At day 16, chick embryos were sacrificed by decapitation. Only embryos with no malformations at day 16 were included in the study. According to the French legislation, approval from an institutional or licensing committee is not required for experiments on the chorioallantoic membrane (CAM) terminated before embryonic developmental day 17.

### ORO staining on HCC frozen sections

Ten-micrometer-thickness sections were made from frozen HCC samples, and fixed in 10% formalin for 10 min at room temperature. After rinsing in water, sections were briefly equilibrated in 60% isopropanol and stained with freshly prepared Oil Red O working solution for 10 min. Excess stain was removed by washing in 60% isopropanol, followed by rinsing in water. Nuclei were counterstained with hematoxylin, and sections were mounted using an aqueous mounting medium. Stained slides were digitized with a Scanscope AT turbo® (Leica Microsystems) slides scanner. % of ORO-positive hepatocytes was determined by a pathologist in a blinded manner.

### Correlation matrix

RNA-seq reads data for hepatocellular carcinoma (HCC) cell lines were extracted and normalized from the CCLE dataset (https://portals.broadinstitute.org/ccle). The selected cell lines were as follow: JHH2 (ACH-000577), KMCH-1 (ACH-001853), SNU475 (ACH-000422), SNU-761 (ACH-000537), SNU-878 (ACH-000686), SNU886 (ACH-000316), SNU-398 (ACH-000221), SNU49 (ACH-000420), SNU182 (ACH-000483), SNU387 (ACH-000478), SNU423 (ACH-000493), PLC-PRF5 (ACH-001318), HEP3B217 (ACH-000625), SK-HEP1 (ACH-000361), HUH1 (ACH-000475), HUH7 (ACH-000480), HLF (ACH-000393), JHH4 (ACH-000476), JHH5 (ACH-000734), JHH6 (ACH-000217), JHH7 (ACH-000848), JHH1 (ACH-000620), and LI7 (ACH-000471). Python (version 3.11.9) was used for all data analysis. Pearson correlation coefficients were calculated to identify significant relationships of expression between RelB and other genes. Only correlations with an absolute value greater than or equal to 0.3 were considered significant. Genes correlated with RelB were functionally annotated using Gene Ontology (GO) terms obtained from the Gene Ontology database, focusing on terms related to «lipase».

### K-means clustering

Using the CCLE dataset for HCC cell lines as above, unsupervised Principal Component Analysis (PCA) was performed (scikit-learn 1.5.1) with 2 outliers removed (SNU-761/ACH-000537 and PLC-PRF5/ACH-001318). HCC cell lines were clustered using the unsupervised *K*-Means (scikit-learn 1.5.1) method based on their «lipase» genes expression profiles, and two independent clusters were identified. Mann–Whitney *U* tests were conducted to compare the expression levels of selected genes between the two clusters identified by *K*-Means clustering. RelB expression was significantly different between the two clusters, named High-RelB vs Low-RelB expressing HCC cell line clusters. Statistical significance was assessed with a *p*-value threshold of <0.05.

### Determination of RelB gene expression signature in HCC patients

Kaplan-Meier method using Python (version 3.11.9) and lifelines library were first used to define a subset of high-RelB HCC patients with a poor prognosis impact on disease specific survival (DSS) and overall survival (OS) from RNAseq dataset of the TCGA cohort of HCC patients (TCGA-LIHC, *n* = 365), and confirmed on a validation cohort (GSE 36376, *n* = 239). Next, using the heatmaply v1.5.0 R package, heatmap was generated to compare gene expression levels defining two distinct subgroups corresponding to high-RelB and low-RelB expression HCC patients. The heatmap includes genes differentially expressed with a fold change ≥2 and a *p* value < 0.05.

### RNAseq

Total RNAs were extracted from high RelB and low RelB Huh7 cells (three independent biological replicates per condition) using TRIzol (Invitrogen, 15596026) according to the manufacturer’s recommendations, followed by separation with chloroform, precipitation with isopropanol, and washing with 80% ethanol. The RNA was then resuspended in RNase-free water and quantified with a Nanodrop 2000 spectrophotometer. RNA purity and integrity was evaluated by the GenomeEast platform a member of the ‘France Génomique’ consortium (Illkirch, France) with the Agilent 2100 Bioanalyzer (Agilent Technologies). Only samples with RNA purity ratio 28S/18S ≥ 1.6 and RNA integrity number (RIN) ≥ 7 were used for library preparation. RNA sequencing librairies and sequencing were performed by the GenomeEast platform. Briefly, RNA-Seq libraries were generated from 600 ng of total RNA using TruSeq Stranded mRNA LT Sample Preparation Kit (Illumina, San Diego, CA), according to manufacturer’s instructions (TruSeq Stranded mRNA Sample Preparation Guide - PN 15031047: Rev.E Oct 2013). The final cDNA libraries were checked for quality and quantified using capillary electrophoresis with 2100 Bioanalyzer (Agilent Technologies France, Les Ulis) and further sequenced on a HiSeq 4000 sequencer (Illumina) as single-end 50 base reads following Illumina’s instructions.

Reads were preprocessed using cutadapt 1.10 (Martin, 2011) in order to remove adaptors and low-quality sequences and reads shorter than 40 bp were removed for further analysis. Remaining reads were mapped to Homo sapiens rRNA and ERCC Spike-In sequences using bowtie 2.2.8 and reads mapped to those sequences were removed for further analysis. Remaining reads were aligned to hg38 assembly of Homo sapiens with STAR 2.5.3a. Gene quantification was performed with htseq-count 0.6.1p1, using “union” mode and Ensembl 94 annotations. Differential gene expression analysis was performed using DESeq2 1.16.1. Bioconductor R package on previously obtained counts (with default options) Genes with an adjusted *P* value < 0.05 were labelled significant. Enrichment analysis from differentially expressed genes has been performed using the enrichGO function from clusterProfiler package v3.16.1. *P*-values were adjusted for multiple testing using the Benjamini and Hochberg method. The RNAseq data have been deposited in the Sequence Read Archive (SRA) at the NCBI under BioProject accession number PRJNA1434356.

### Metabolomics by NMR

NMR sample preparation. Exponentially growing high-RelB and low-RelB Huh7 cells were seeded in a total volume of 10 ml in 100 cm^2^ petri dish (total 5 × 10^6^ cells/condition). To address the reproducibility of the experiment, 10 individual samples of dry cell pellets were prepared. Dry cell pellets were treated with 400 µL of ice-cold methanol and 65 µL of ice-cold water. A volume of 200 µL of ice-cold chloroform, then 200 µL of ice-cold water and finally 200 µL of ice-cold chloroform were further added. The samples were then left on ice for 15 min, and centrifugated at 2000 rpm for 15 min. The upper methanol/water phase (containing polar metabolites) and the lower chloroform phase (containing lipophilic compounds) were transferred into separate glass vials. Solvents were removed under a stream of nitrogen for 30 min. The water phases were put in the freeze dryer one night. The dry water phase samples were resuspended in 580 µL of NMR buffer (100 mM sodium phosphate buffer pH 7.4, in D_2_O, with 3.57 mM TSP and 6 mM NaN_3_) to obtain the polar phase extract. The lipophilic phases were resuspended in 600 µL of deuterated NMR solvent (2:1 mixture of pure CDCl_3_ and CDCl_3_ containing 0.03% of TMS, to obtain a final solution of 0.01% of TMS) and then vortexed. After centrifugation (5 min), 580 µL of the supernatant was directly transferred into an NMR tube to prepare the organic phase extract.

Lipophilic phase extraction of CAM tumors. The frozen biopsies (around 15–20 mg), placed in a 2 mL round-bottom Eppendorf tube, were mixed with CH_3_OH/H_2_O (0.4 mL/0.065 mL), and two grinding stainless steel beads were added inside (Qiagen) to grind them (2 min at 20 Hz with a ball tissue lyser device). Supernatants were then recovered and placed in new Eppendorf tubes, 200 μL of CHCl_3_ was added, and the tubes were vortexed. Then CHCl_3_ and H_2_O (200 μL each) were added, and the tubes were vortexed again to allow for solvent extraction. The tubes were placed at 4 °C for at least 15 min to accelerate the extraction then centrifuged for 15 min at 1000 × *g*. The lipophilic phases were isolated and the residual CHCl_3_ solvent was evaporated under N_2_ gas using a sample concentrator Stuart SBHCONC/1 (15 kPa bar at 18.5 cm). Next, the lipophilic phases were resuspended in 600 µL of deuterated NMR solvent (2:1 mixture of pure CDCl_3_ and CDCl_3_ containing 0.03% of TMS, to obtain a final solution of 0.01% of TMS) and then vortexed. After centrifugation (5 min), 580 µL of the supernatant was directly transferred into an NMR tube to prepare the organic phase extract of tumors.

NMR data acquisition. All samples were measured on a 500 MHz Bruker Avance III spectrometer equipped with a SampleXpress automation sample changer and a cryogenic 5 mm ^13^C/^1^H dual probe with Z-gradient. The endometabolome samples were acquired using the classical experiment used for the metabolomic analysis of cell or tissue extracts by NMR [58]. 1D-CPMG pulse sequence with presaturation and 1D 1H experiment were performed for the aqueous phase extract and the organic phase extract, respectively. The operating software was TopSpin 3.1 1 (Bruker BioSpin, France).

Metabolite data processing and metabolite identification. For each NMR spectrum, the standard processing protocol was applied, including Fourier transform, phase correction, baseline correction, and calibration. For metabolite identification, ^1^H NMR spectra were analyzed using Chenomx NMR Suite 8.0 (Chenomx Inc.). Metabolite identification was performed using the spectral reference library and fitting peak intensity and location to the observed signals. The identification of compounds found in the organic phase was based on the publication of Amiel et al. [59].

The processing of raw data was carried out using NMRProcflow, including baseline correction, chemical shift calibration, data alignment, and variable-sized bucketing. For all data sets, a threshold corresponding to a signal-to-noise ratio of 3 was applied.

Statistical Analysis. A constant sum normalization was applied for all samples. Subsequently, we used SIMCA 15 software (Umetrics) to perform principal component analysis (PCA) or orthogonal projections to latent structure discriminant analysis (OPLSDA) to discriminate cancer cell line classes. Pareto scaling was used in both analyses. MetaboAnalyst 6.0 was used to perform *t*-tests and determine *p*-values with FDR correction and to generate box plots (https://www.metaboanalyst.ca/).

### Proteomics and mass spectrometry analysis

Sample preparation: 20 µg of samples were incubated overnight at −20 °C with 50 µl of 100% Methanol buffer (MeOH, 0.1 M ammonium acetate, glacial). Proteins were precipitated by centrifugation at 14,000 × *g* for 15 min at −5 °C, and the pellets were washed once with 100 µl of 80% Methanol buffer (MeOH/H_2_O, 0.1 M ammonium acetate, glacial). Dried protein pellets were resuspended in 5 µL of Urea buffer (4 M urea, 100 mM ammonium bicarbonate), and cysteine residues were reduced with 5 µl of 10 mM dithiothreitol at 57 °C for 30 min. Cysteine residues were then alkylated by adding 2 µl of 55 mM iodoacetamide for 30 min at room temperature. Following alkylation, the urea concentration was diluted to 1 M with 100 mM ammonium bicarbonate, and proteins were digested overnight at 37 °C with 0.4 μg of trypsin/LysC (Promega) per sample. Samples were subsequently desalted using homemade C18 StageTips. Peptides were eluted using 40% acetonitrile buffer (MeCN/H_2_O, 0.1% formic acid), vacuum concentrated to dryness and resuspending in 0.1% formic acid supplemented with 0.015% n-dodecyl-β-D-maltoside prior to liquid chromatography–tandem mass spectrometry (LC–MS/MS) analysis.

LC-MS/MS analysis: Liquid chromatography (LC) was performed with a Vanquish Neo LC system (Thermo Scientific) coupled to an Orbitrap Astral mass spectrometer (Thermo Scientific), interfaced by a Nanospray Flex ion source (Thermo Scientific). Peptides were initially trapped on a C18 column (75 μm inner diameter × 2 cm; nanoViper Acclaim PepMap^TM^ 100, Thermo Scientific) using buffer A (H_2_O with 0.1% formic acid). Peptide separation was then performed on a 50 cml̥× 75 μm C18 column (nanoViper Acclaim PepMap^TM^ RSLC, 2 μm, 100 Å, Thermo Scientific) maintained at 50 °C, using a linear gradient from 2% to 28% buffer B (MeCN with 0.1% formic acid) at a flow rate of 300 nL/min over 105 min. MS full scans were acquired in centroid mode in the Orbitrap mass analyzer over an *m/z* range of 380–980 at a resolution of 240,000 (at *m/z* 200), with a normalized auto gain control (AGC) target set at 500% and a maximum injection time of 5 ms. Data independent acquisition (DIA) scans were acquired in centroid mode in the Astral analyzer over the same *m/z* range (380–980), using isolation windows width of 2 Da (without overlap), maximum injection time of 3 ms, and normalized AGC target of 500%. Fragmentation was performed by higher-energy collisional dissociation (HCD) with a normalized collision energy of 25%.

Data processing: For identification, the data were searched against the *Homo sapiens* (UP000005640) Uniprot database using Pulsar search engine through Spectronaut® 20 (Biognosys) by directDIA^TM^ analysis using default search settings. Enzyme specificity was set to trypsin, and a maximum of two missed cleavages was allowed. Carbamidomethylation of cysteines was set as a fixed modification, while N-terminal acetylation and oxidation of methionine were set as variable modifications. The resulting files were further processed using v3.10.0 myProMS [https://github.com/bioinfo-pf-curie/myproms][60].

For protein quantification, ion XICs (sum of fragment peak areas) from proteotypic peptides shared between compared conditions (TopN) were used, with missed cleavages allowed. Median and scale normalization at peptide level was applied on the total signal to correct the XICs for each biological replicate (*n* = 5). To evaluate the statistical significance of the change in protein abundance, a linear model (adjusted on peptides and biological replicates) was performed, and a two sided *t*-test was apply on the fold change estimated by the model. The *p*-values were then adjusted using the Benjamini–Hochberg FDR procedure.

The mass spectrometry proteomics raw data have been deposited to the ProteomeXchange Consortium via the PRIDE [61] partner repository with the dataset identifier PXD074627.

### Statistical analysis

Kaplan-Meier estimates of overall survival were performed by using the log-rank test (Mantel–Cox). All statistical analyses were performed with Python software (https://www.python.org/, version 3.11.9) or GraphPad Prism software (version 10.1.2). Statistical analysis by Student's *t*-test or Mann–Whitney test. A value of *p* = 0.05 was considered as statistically significant with the following degrees: *p < 0.05; **p < 0.01; ***p < 0.001; ****p < 0.0001.

## Supplementary information

Original data

Supplemental Figure 1

Supplemental Figure 2

Supplemental Figure 3

Supplemental Table 1

Supplemental Table 2

Supplemental Table 3

## Data Availability

The mass spectrometry proteomics raw data have been deposited to the ProteomeXchange Consortium via the PRIDE partner repository with the dataset identifier PXD074627. The RNAseq data have been deposited in the Sequence Read Archive (SRA) at the NCBI under BioProject accession number PRJNA1434356. All other data are available from the corresponding author upon reasonable request.
