# Supplementary material for: The alternative NF-κB RelB subunit is a novel critical sensor of lipid metabolism to promote tumor development
Source: Cell Death Dis. 2026 Jul 4;17(1):830. doi: 10.1038/s41419-026-09064-7 (PMC13612402; doi:10.1038/s41419-026-09064-7)

Figure 3 A

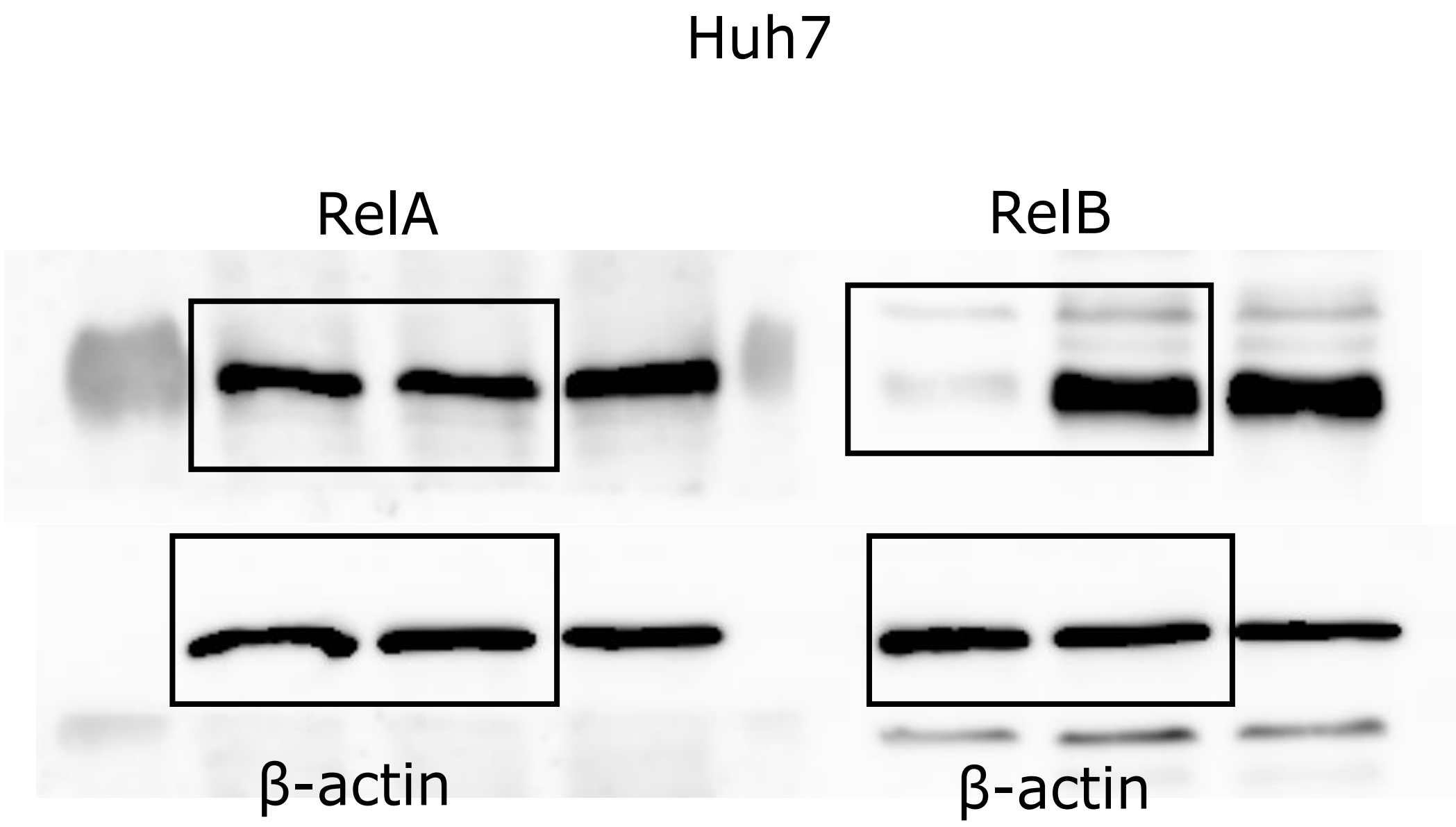

Figure 3 O

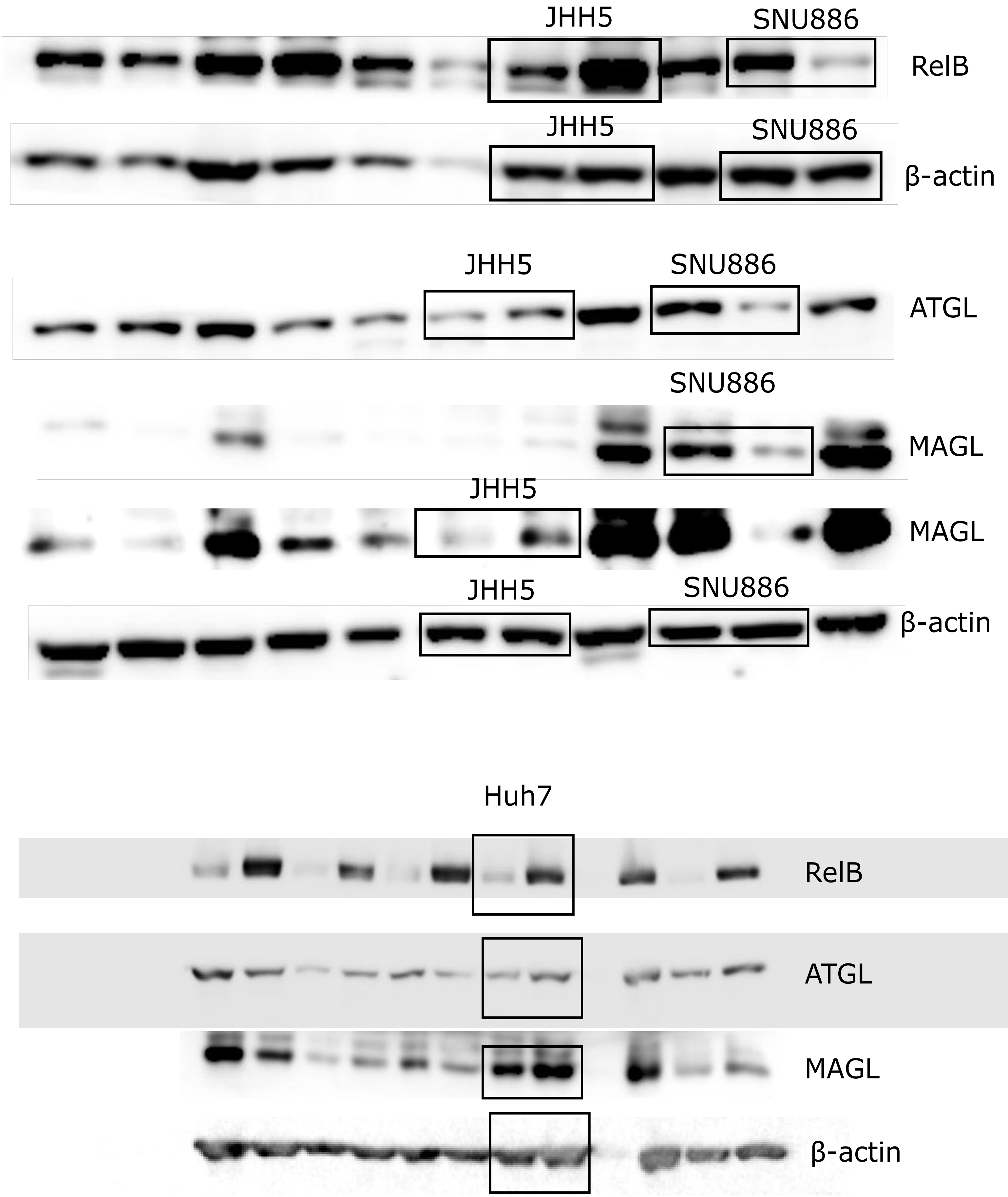
JHH5
SNU886
RelB
JHH5
SNU886
β-actin
JHH5
SNU886
ATGL
SNU886
MAGL
JHH5
MAGL
JHH5
SNU886
β-actin
Huh7
RelB
ATGL
MAGL
β-actin

Figure 4 G

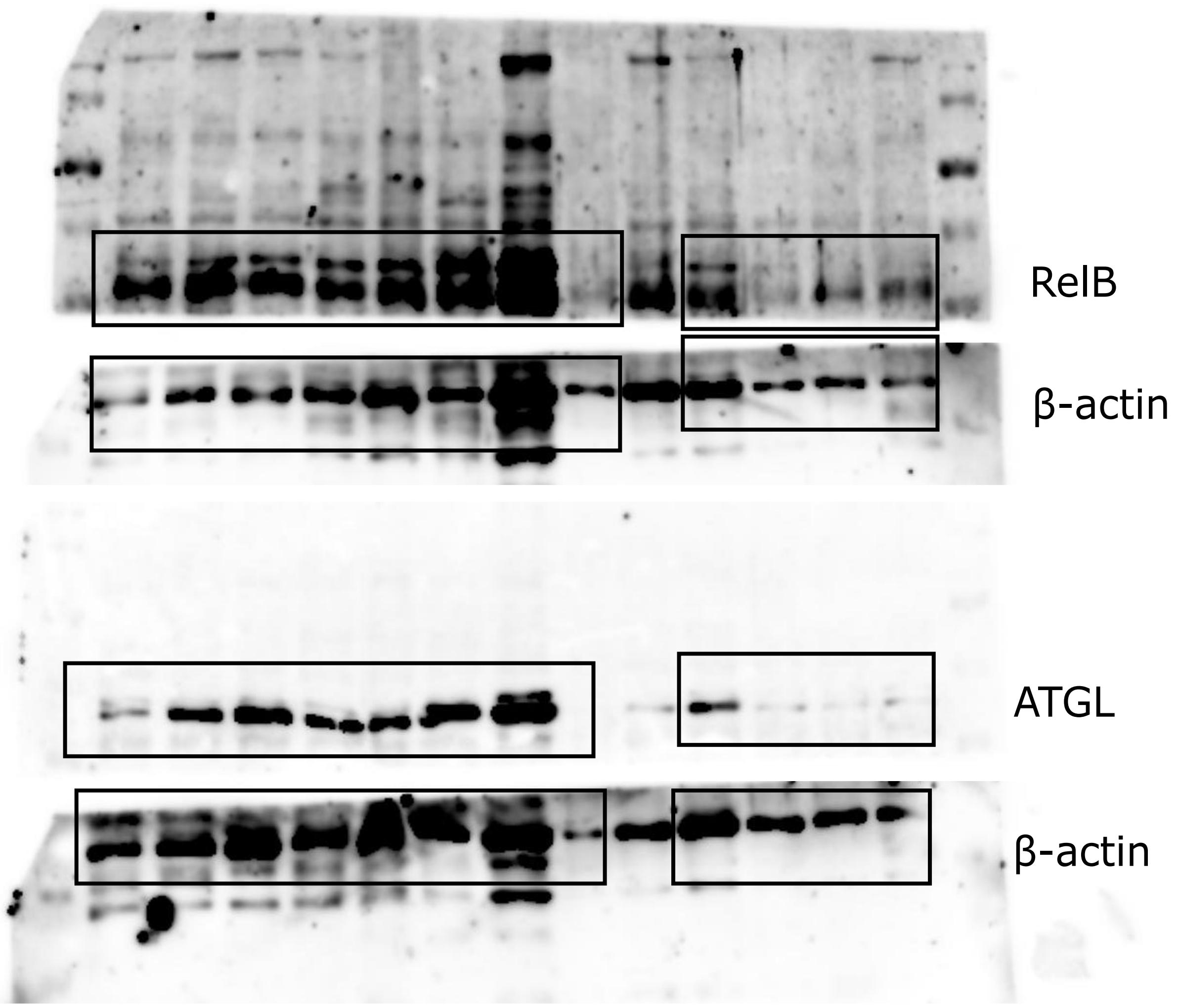

Supplemental figure 3 A

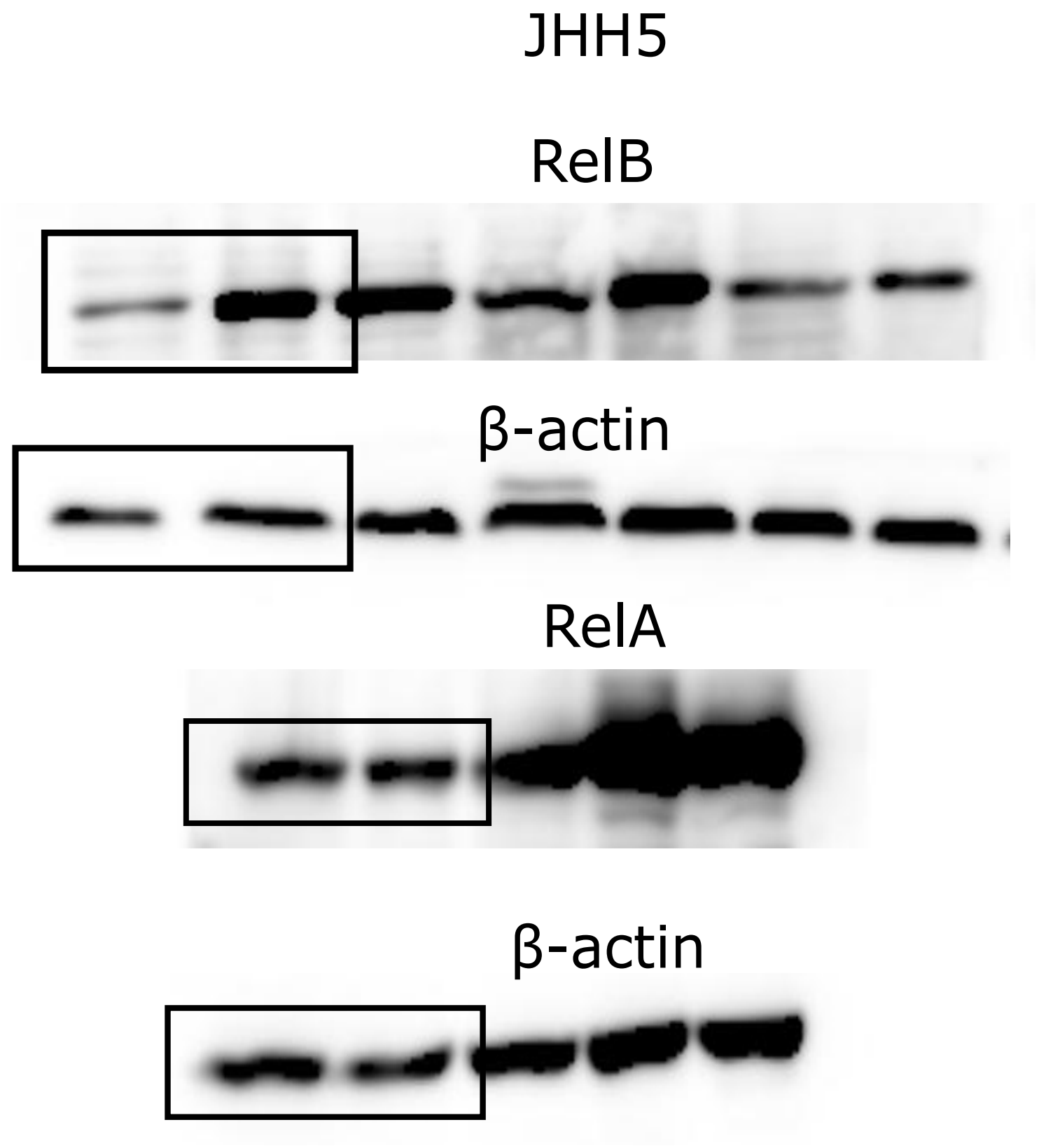

Supplemental figure 3 E

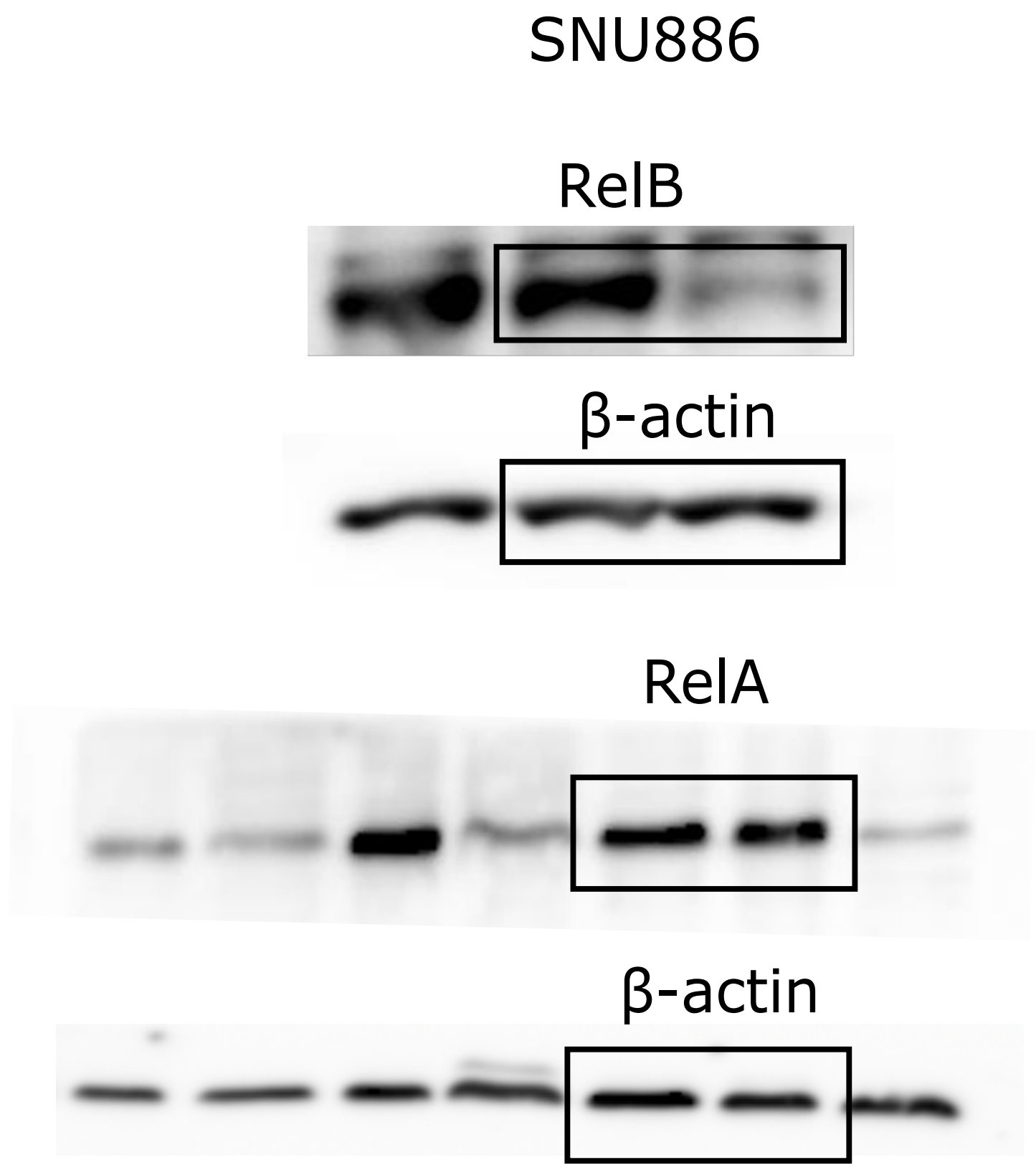

Supplement: Supplementary file 1 [file 41419_2026_9064_MOESM1_ESM.pdf]
