# Supplementary material for: The alternative NF-κB RelB subunit is a novel critical sensor of lipid metabolism to promote tumor development
Source: Cell Death Dis. 2026 Jul 4;17(1):830. doi: 10.1038/s41419-026-09064-7 (PMC13612402; doi:10.1038/s41419-026-09064-7)
Supplement: Supplementary file 2 [file 41419_2026_9064_MOESM2_ESM.pdf]

A

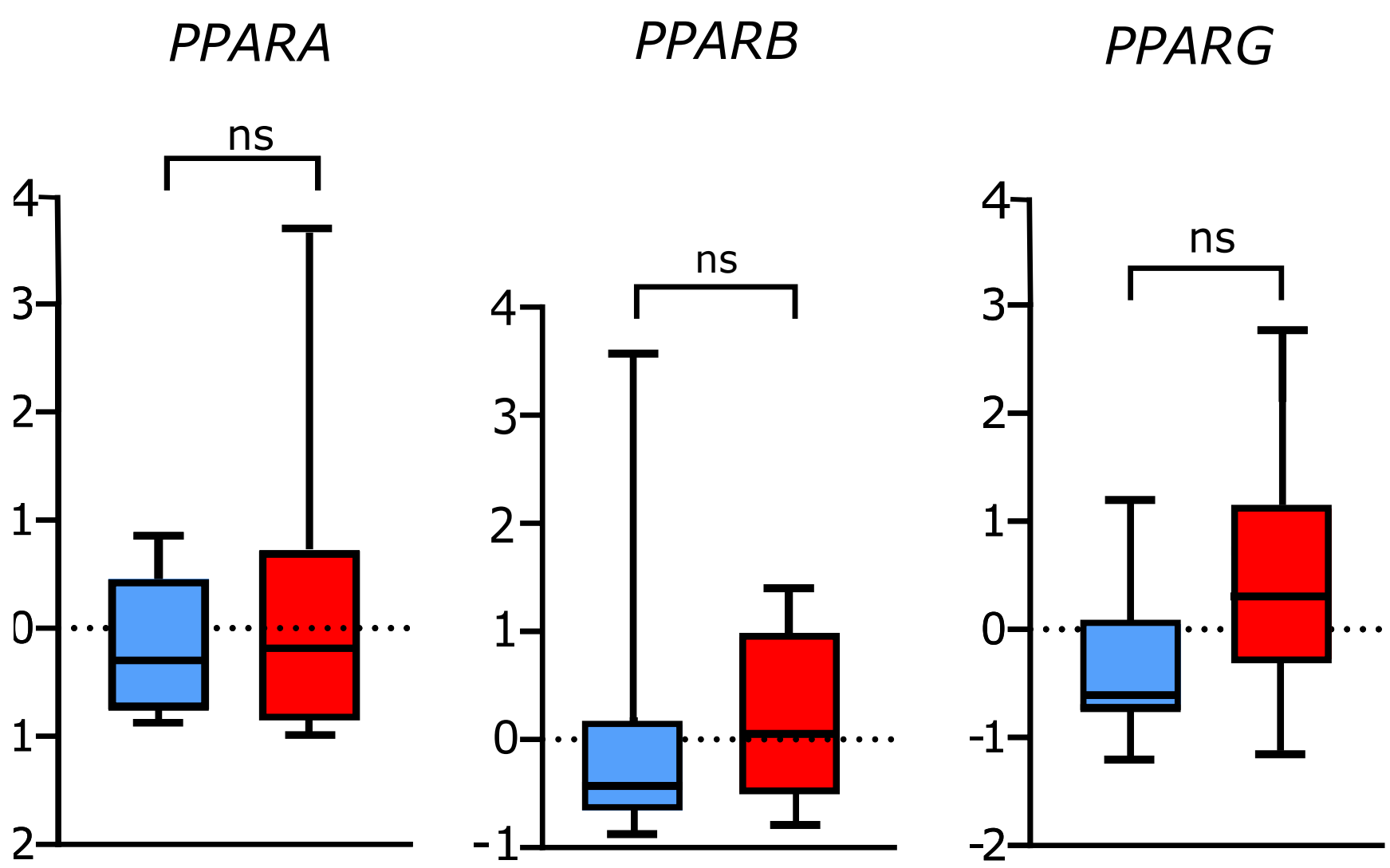

B

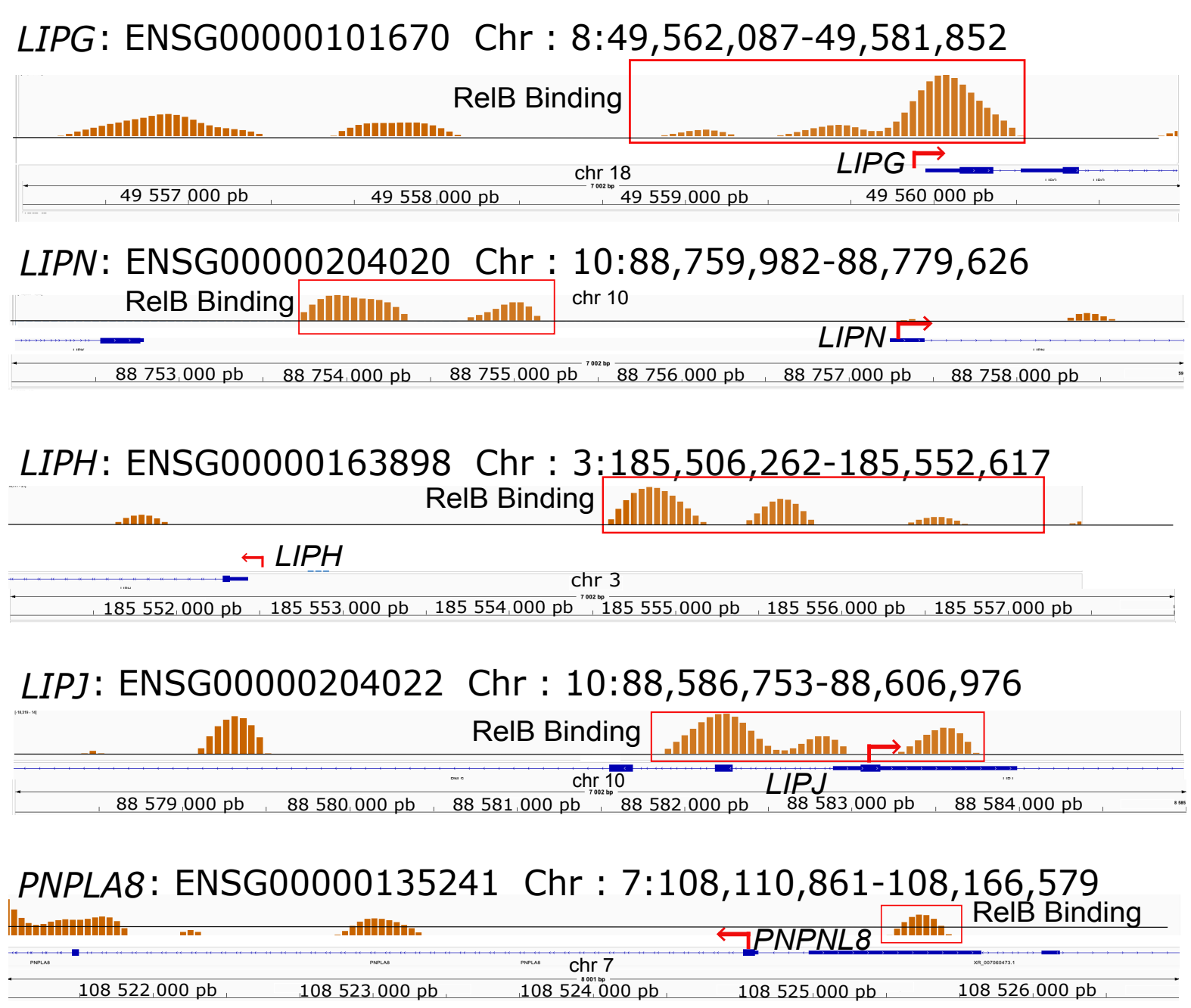

Supplemental Figure 1: **(A)** High-RelB and low-RelB clusters do not exhibit significant difference in *PPARs* expression. Boxplot of *PPARA*, *PPARB* and *PPARG* expression in high-RelB (red) vs low-RelB (blue) clusters, P-values by unpaired t test, ns: not significant, errors bars represent min and max values. **(B)** RelB recruitment to the promoter of *LIPG*, *LIPN*, *LIPJ* and *PNPLA8*. The RelB recruitment is indicated by bars and framed in red. Data from ChiP Atlas, GSE 55105. Visualization by IGV browser.
