# Supplementary material for: The alternative NF-κB RelB subunit is a novel critical sensor of lipid metabolism to promote tumor development
Source: Cell Death Dis. 2026 Jul 4;17(1):830. doi: 10.1038/s41419-026-09064-7 (PMC13612402; doi:10.1038/s41419-026-09064-7)
Supplement: Supplementary file 3 [file 41419_2026_9064_MOESM3_ESM.pdf]

A

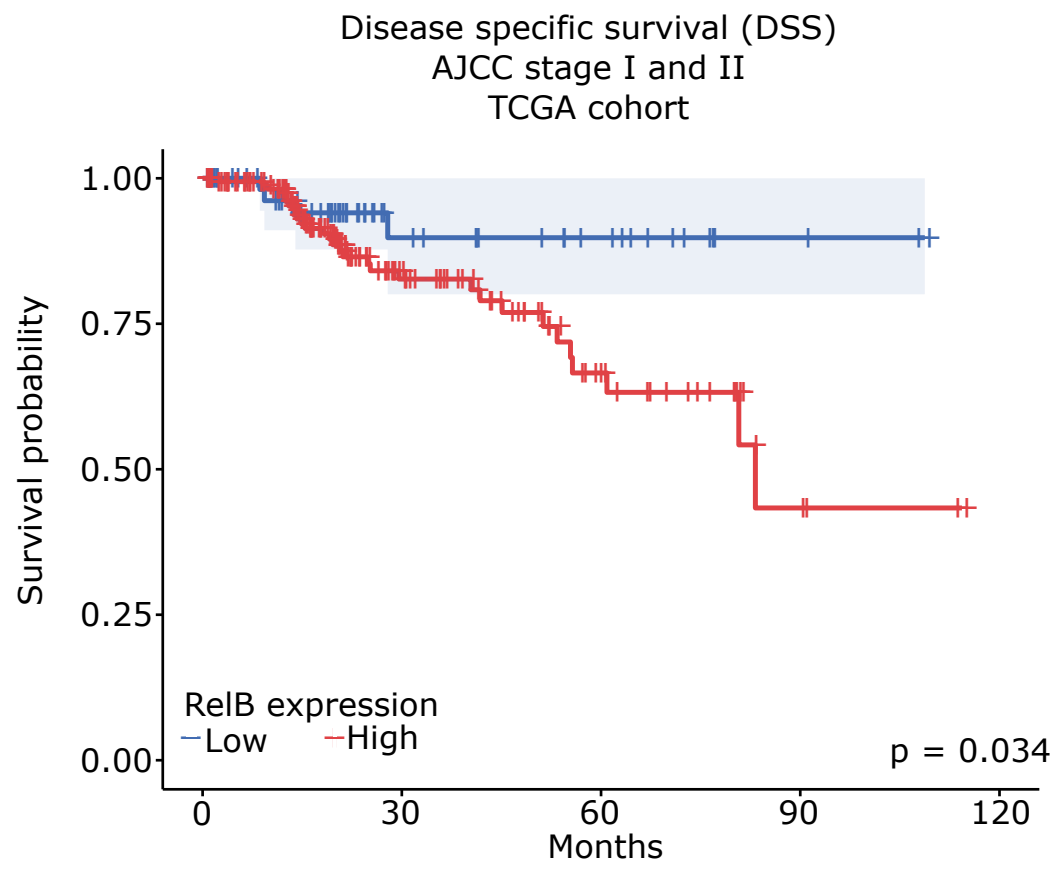

Supplemental Figure 2: **(A)** High RelB expression strongly impacts on early-stage HCC patient outcome (stages I and II defined by the AJCC7th classification). Kaplan-Meier plots of disease specific survival (DSS) according to RelB expression levels (LIHC-TCGA cohort, n= 254)). P value by log-rank (Mantel-Cox) test

Supplemental Figure 2: Becquard. T*, Benatar. C* *and al.*
