# Supplementary material for: The alternative NF-κB RelB subunit is a novel critical sensor of lipid metabolism to promote tumor development
Source: Cell Death Dis. 2026 Jul 4;17(1):830. doi: 10.1038/s41419-026-09064-7 (PMC13612402; doi:10.1038/s41419-026-09064-7)
Supplement: Supplementary file 4 [file 41419_2026_9064_MOESM4_ESM.pdf]

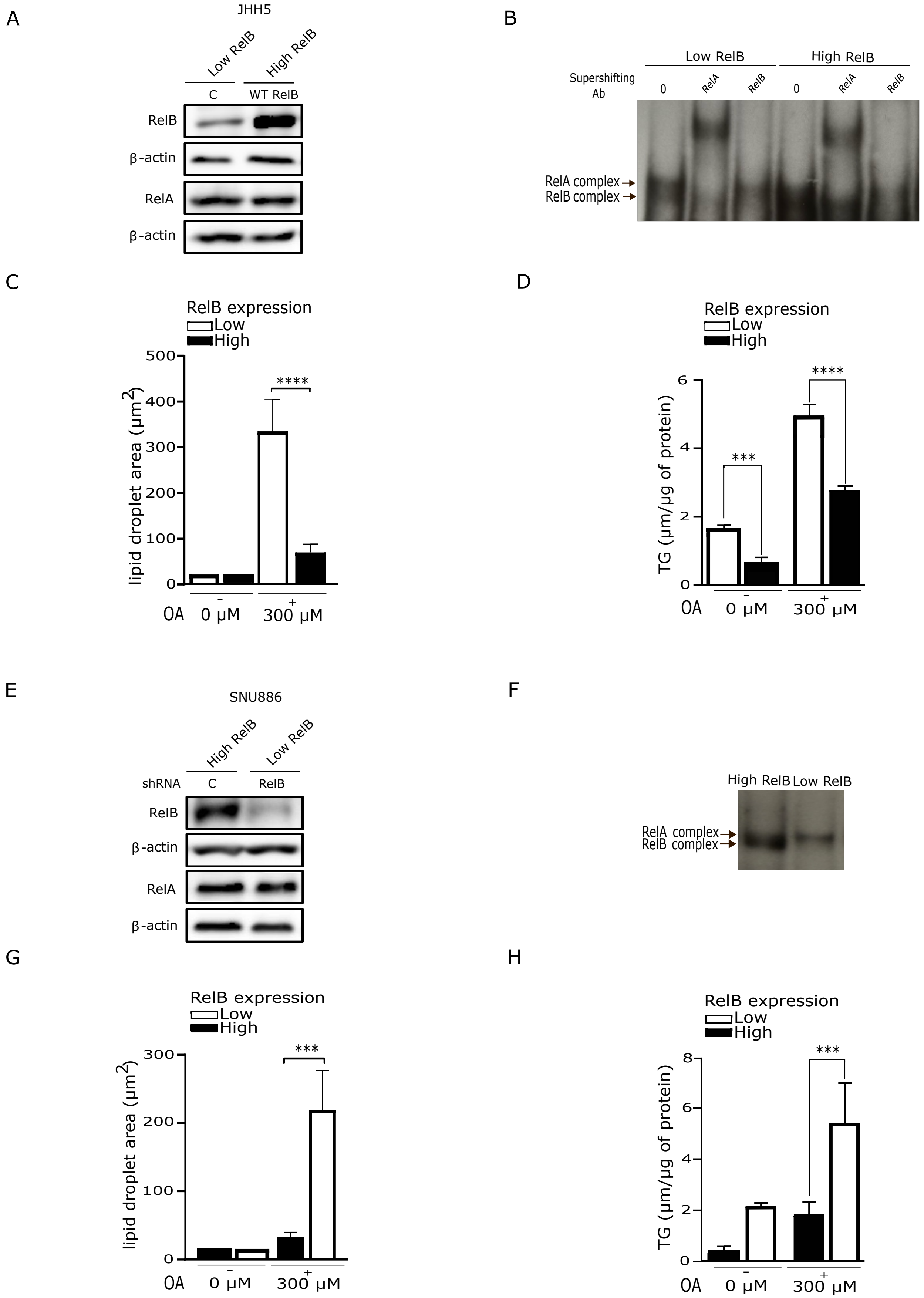

Supplemental Figure 3: Becquard. T*, Benatar. C* *and al.* 1/2

Supplemental Figure 3: **(A)** JHH5 cells stably transduced with lentiviruses encoding either RelB (WT RelB) or a control empty vector (C) were analyzed by immunoblotting for the indicated proteins. High-RelB indicates JHH5 cells expressing high level of ectopic RelB, low-RelB indicates JHH5 cells expressing low endogeneous levels of RelB (in accordance with the transcriptomic analysis of the CCLE dataset, see Figure 1B). **(B)** Whole cell extracts from JHH5 cell lines as described in (A) were analyzed for NF-κB binding activity using EMSA. For supershifts, extracts were incubated with the indicated antibodies before incubation with the labeled probe. RelB- and RelA-containing complexes are indicated. **(C)** Lipid droplet content in high-RelB and low-RelB JHH5 cells treated or not with oleic acid. Magnification: x20. Error bars represent means ± SEM (n=3). P-values by unpaired t test, **** $p<0.0001$. **(D)** Total TAG content of high-RelB and low-RelB JHH5 cells treated with exogenous oleic acid for 16h was measured after lipid extraction by the Folch method. Error bars represent means ± SEM (n = 3). P-values by Mann Whitney tests, *** $p<0.001$, **** $p<0.0001$. **(E)** SNU886 cells stably transduced with lentiviruses encoding either a shRNA targeting RelB or a scrambled control (C) were analyzed by immunoblotting for the indicated proteins. High-RelB indicates SNU886 cells expressing high endogeneous levels of RelB (in accordance with the transcriptomic analysis of the CCLE dataset, see Figure 1B), low-RelB indicates SNU886 cells expressing low levels of RelB upon endogeneous RelB expression knockdown. **(F)** Whole cell extracts from SNU886 cell lines as described in (E) were analyzed for NF-κB binding activity using EMSA. RelB- and RelA-containing complexes are indicated. **(G)** Lipid droplet content in high-RelB and low-RelB SNU886 cells treated or not with oleic acid. Magnification: x20. Error bars represent means ± SEM (n=3). P-values by unpaired t test, *** $p<0.001$. **(H)** Total TAG content of high-RelB and low-RelB SNU886 cells treated with exogenous oleic acid for 16h was measured after lipid extraction by the Folch method. Error bars represent means ± SEM (n = 3). P-values by Mann Whitney tests, *** $p<0.001$.
