# Supplementary material for: The alternative NF-κB RelB subunit is a novel critical sensor of lipid metabolism to promote tumor development
Source: Cell Death Dis. 2026 Jul 4;17(1):830. doi: 10.1038/s41419-026-09064-7 (PMC13612402; doi:10.1038/s41419-026-09064-7)
Supplement: Supplementary file 5 [file 41419_2026_9064_MOESM5_ESM.pdf]

Genes	Pearson correlation	Genes	Pearson correlation
RELB	1	C5AR1	0,43091266
GPR157	0,75282039	SMPD1	0,42933926
LIPH	0,73231228	ABHD16A	0,42744824
P2RY4	0,70471155	PNPLA2	0,42167935
DRD5	0,68021345	ANG	0,41994241
HTR2A	0,67738331	PLD2	0,41950153
NTSR2	0,67671052	FAAH	0,41899403
AGTR1	0,64613665	ORAI1	0,40278988
CHRM1	0,64007061	DAGLB	0,39899799
C5AR2	0,63432161	PRKN	0,39676985
PNPLA7	0,63063886	ANXA4	0,38611418
EDN1	0,59594056	PLBD1	0,36354606
LIPE	0,59150592	PLCE1	0,36030636
PLD1	0,57185741	PLCD3	0,35292349
BDKRB2	0,56741828	LIPC	0,35134765
PDIA3	0,55639654	ANXA8	0,34522369
VIP	0,54528918	GDPD1	0,34475386
HCRT	0,53211074	GPR4	0,34320873
PLBD2	0,52711534	STXBP1	0,32164737
DGKQ	0,52530504	PITPNM3	0,31670895
LRPAP1	0,50776887	LPAR2	0,3142329
PRKD2	0,49570955	PROCA1	0,30654305
PNPLA6	0,49517214	TRHR	0,30451418
AGT	0,48791412	ANGPTL4	0,30173302
GRM5	0,48677321	PLCB4	-0,30131882
LIPK	0,47304153	OPRM1	-0,31081403
ABHD3	0,47037397	SYK	-0,32290362
S1PR4	0,45904362	PLA2G4A	-0,32530215
PLA2G4C	0,45288565	HTR2C	-0,32668194
NR1H2	0,44788409	LRP1	-0,34145851
PTAFR	0,44664113	PNLIPRP2	-0,36591553
LIPN	0,4383101	LMNB1	-0,38334983
PNPLA4	0,43155961		

Supplemental Table 1 : Pearson correlation values between mRNA expression levels of RelB and sixty-four lipases from the CCLE HCC cell line dataset. (related to Figure 1A).
