# Supplementary material for: The alternative NF-κB RelB subunit is a novel critical sensor of lipid metabolism to promote tumor development
Source: Cell Death Dis. 2026 Jul 4;17(1):830. doi: 10.1038/s41419-026-09064-7 (PMC13612402; doi:10.1038/s41419-026-09064-7)
Supplement: Supplementary file 6 [file 41419_2026_9064_MOESM6_ESM.pdf]

AASS
ABCB11
ABCC9
ABCG2
ABHD1
ACAN
ACAP1
ACE2
ACSL4
ACSL5
ACSL6
ACSM3
ACSS3
ACTG1P25
ACTN2
ADAM28
ADAM6
ADAM8
ADAMDEC1
ADAMTS3
ADAP1
ADCY1
ADGRG3
ADH1B
ADH4
ADM2
ADORA1
ADRA1A
ADRB1
ADTRP
AFAP1-AS1
AFP
AIM2
AKAP12
AKR1B10
ALOX5
ALOX5AP
AMACR
ANKRD37
ANO3
ANO9
ANXA13
AOAH
APCDD1
APOBEC3D
APOBEC3G
AQP6
AQP9
AR
ARHGAP44
ARHGAP8/PRR5-ARHGAP8
ARHGAP9
ARNT2
ARNTL2
ARPC1B
ARSJ
ASAH2
ASB4
ASCL1
ASNS
ASPA
ASRGL1
ATP1A2
ATP6V0D2
AURKB
AVIL
AVPR1A
AXIN2
B3GNT3
BBOX1
BCAN
BCAS1
BCL11B
BCL2A1
BCL2L14
BCRP3
BICC1
BIRC3
BIRC5
BTK
BTLA
BTNL9
C11orf45
C12orf75
C15orf48
C16orf89
C16orf96
C1orf116
C1QTNF3
C2CD4A
C3orf52
C5orf46
C6orf132
C9orf152
CA14
CA4
CA5B
CABP4
CACNA2D3
CALHM6
CAMK2A
CAMK4
CAPG
CARD11
CARD14
CARD9
CARMIL2
CASQ2
CAVIN2
CBLN4
CCDC170
CCDC183
CCDC88B
CCL13
CCL17
CCL2
CCL20
CCL22
CCL5
CCR4
CCR5
CCR6
CCR7
CCT6B
CD1C
CD1E
CD2
CD24
CD27
CD300C
CD300LB
CD300LF
CD300LG
CD36
CD37
CD38
CD3D
CD3E
CD3G
CD40LG
CD48
CD5
CD52
CD53
CD6
CD72
CD79A
CD86
CD8A
CD8B
CD96
CDC14C
CDC42BPG
CDCP1
CDH15
CELF6
CEP55
CERS1
CES4A
CES5A
CFAP57
CHRDL2
CHST1
CHST11
CKMT2
CLDN4
CLEC5A
CLEC7A
CLECL1
CLGN
CLIC5
CLSTN2
CNDP1
CNKSR2
CNNM1
CNTNAP3
COL24A1
COL25A1
COL7A1
COL9A2
CORIN
CORO1A
CRACD
CRACR2B
CRTAM
CRYBB2
CSF2RA
CSF2RB
CSF3R
CST7
CTH
CTHRC1
CTLA4
CTNNA2
CTSK
CTSS
CXCL1
CXCL3
CXCL6

CXCL8
CXCR1
CXCR3
CXCR6
CYBB
CYP1A1
CYP1A2
CYP26A1
CYP2A6
CYP2B7P
CYP2E1
CYP2S1
CYP39A1
CYP3A4
CYP3A43
CYP4A22
CYP8B1
CYTOR
DAAM2
DAB1
DAPP1
DBN1
DCANP1
DDI2
DDR1
DEF6
DEPDC1B
DHDH
DIAPH3
DKK3
DMGDH
DMKN
DNAJB3
DNAJC5B
DNMT3L
DOCK2
DOK2
DRD1
DSG1
DUSP4
DUSP5
DUSP9
DYNC1I1
DYNLT5
DZIP1L
ECM2
EDAR
EFNA5
EGLN3
EHHADH
ELF4
ELFN1
EMB
EMID1
ENTPD2
EOMES
EPHB2
EPHB3
EPHB6
EPO
ESR1
ESRRG
ETV4
ETV7
EVC2
EXPH5
FABP4
FAM13C
FAM169A
FAM180A
FAM186B
FAM225A
FAM225B
FAM27C
FAM27C
FASLG
FBXO41
FCER1G
FCGBP
FCGR1A
FCGR1B
FCGR1CP
FCMR
FCRL5
FER1L4
FERMT3
FFAR2
FFAR3
FGD3
FGFR2
FHDC1
FHL5
FHOD3
FMNL1
FMO2
FNDC1
FNDC5
FOLH1B
FOXA3
FOXP3
FOXS1
FPR1
FPR2
FRMD3
FSIP1
FSTL3
FUT2
FUT4
FUT6
FUT7
FYB1
FZD2
FZD7
G6PD
GABARAPL3
GAL3ST1
GAL3ST4
GALNT15
GALNT18
GALNT6
GAPT
GAS7
GCK
GCNT3
GCSAM
GDPD3
GFPT2
GJB3
GLI1
GLUL
GLYAT
GMPR
GNAZ
GNB3
GOLGA6A
GP1BA
GPLD1
GPR132
GPR171
GPR18
GPR35
GPR37L1
GPR55
GPR68
GPR84
GPX2
GREB1
GRIN2D
GRPR
GSDMA
GSTM1
GSTM2
GSTM2P1
GXYLT2
GYS2
GZMA
GZMK
GZMM
H19
H2AC11
HAND2
HAPLN3
HAPLN4
HBB
HCAR2
HCAR3
HCK
HEPN1
HID1
HKDC1
HLA-DOA
HLA-DOB
HLA-DQA1
HLA-DQA2
HLA-DQB1
HLA-DQB2
HLF
HMGA1
HNF1B
HORMAD2
HOXD1
HPSE
HSD11B1
HSD17B13
HSH2D
HSPA6
HTR1D
HTR2B
HTRA3
ICAM1
ICOS
IER3
IFI27
IGDCC4
IGF2BP3
IGSF1
IGSF10
IGSF3
IKBKE
IL12RB1

IL18
IL1B
IL21R
IL2RA
IL2RG
IL32
IL4I1
INE2
INHBE
IQCA1
IQCH
IRX5
ITGA3
ITGAM
ITGAX
ITGB2
ITGB4
ITK
ITLN2
ITPR3
JAK3
JAKMIP1
JAKMIP2
JAML
JCHAIN
JSRP1
KAAG1
KAZN
KBTBD11
KCNB1
KCND3
KCNE5
KCNF1
KCNH3
KCNH4
KCNJ11
KCNK17
KCTD17
KIAA1549L
KIF1A
KIF3C
KLC3
KRT19
KRT23
KRT80
KSR2
LAD1
LAG3
LAIR2
LAMA3
LAMP3
LAPTM5
LAX1
LCK
LCN2
LCP1
LDLRAD1
LEFTY1
LGALS2
LGALS9
LGI3
LGR5
LIF
LILRA4
LILRB4
LIMD2
LINC00092
LINC01018
LINC01124
LINC01512
LINGO4
LIPE
LIPH
LMOD1
LMTK3
LOC100130331
LOC100131496
LOC606724
LOXHD1
LOXL4
LPAR2
LPL
LRP12
LRP8
LRRC1
LRRC39
LSP1
LSP1P5
LTA
LTB
LY6E
LY86
LY9
LY96
LYPD6
MACIR
MADCAM1
MAL2
MAMLD1
MAP4K1
MAPK13
MARCHF3
MCF2L2
MCHR1
MCOLN2
MDGA1
MEI1
MELTF
MERTK
MEX3A
MFAP2
MFSD10
MIR155HG
MLANA
MME
MMP25
MMP7
MMP9
MNDA
MOGAT2
MPDZ
MPZ
MROH7
MSC
MSI1
MST1R
MSTN
MTCL1
MX2
MYB
MYBL2
MYH3
MYH7B
MYO16
MYO1G
MYO7B
MYRIP
NAALADL1
NAPSB
NAT2
NCEH1
NCF1
NCF1C
NCF2
NCKAP1L
NCR3
NDRG4
NDUFA4L2
NECAB2
NEURL3
NFE2L3
NFKBIE
NKAPL
NKD1
NLRP14
NNMT
NOTUM
NPW
NQO1
NTRK2
NXPH4
OAT
OPRPN
OSCAR
OSM
OSR2
OXCT1
P2RX5
P2RY10
P2RY6
PACRG
PAFAH1B3
PAK6
PAPPA2
PARVG
PBX4
PCDH20
PCDHB6
PCED1B
PCED1B-AS1
PDCD1
PDE11A
PDE9A
PDGFRL
PDK4
PDZD7
PGM5
PHLDA2
PHYHIP
PIK3CD
PIMREG
PITX1
PIWIL4
PKIB
PKM
PLA2G2D
PLAUR
PLBD1
PLD4
PLEKHA4

PLEKHB1	RBM24	SHISA2	SOX9
PLEKHN1	RBP1	SHISAL2A	SP140
PLIN1	RECQL4	SHOX2	SP5
PLIN4	REEP2	SIGLEC10	SPARCL1
PLPP2	RELB	SIGLEC8	SPATA18
PLPP7	REN	SIRPG	SPATC1L
PLPPR1	RGL4	SIT1	SPDYC
PLXNA4	RGS10	SIX2	SPHK1
PLXNB3	RGS16	SLA2	SPIB
PMEL	RGS4	SLAMF1	SPINK1
PMEPA1	RHBDL2	SLAMF6	SPINT1
POF1B	RHBDL3	SLAMF7	SPINT2
POU2AF1	RHBG	SLAMF8	SPN
PRCD	RHCG	SLC12A2	SPP1
PRKCB	RHOBTB1	SLC13A3	SRD5A2
PRKCQ	RHOF	SLC16A1	SRSF12
PRKN	RHOH	SLC16A11	SSPOP
PROM1	RHOV	SLC16A12	SSTR2
PROM2	RIN1	SLC16A3	ST8SIA1
PRR5L	RNF125	SLC1A2	STAT4
PRR7	RPL39L	SLC1A5	STEAP4
PSTPIP1	RSPH1	SLC1A7	STPG3
PTAFR	RSPO3	SLC25A47	STRA6
PTCH2	RUNDC3B	SLC26A3	STUM
PTCHD4	RUNX3	SLC2A1	SULT1B1
PTGES	S100A11	SLC2A4	SUSD4
PTGFR	S100A14	SLC2A6	SYK
PTGS1	S100A2	SLC30A3	SYN1
PTPN22	S100A4	SLC34A1	SYT1
PTPN7	S100B	SLC38A11	TAGAP
PTPRC	S100P	SLC45A2	TAS1R1
PTPRCAP	S1PR2	SLC45A4	TBC1D10C
PTPRN2	S1PR4	SLC4A11	TBX3
PTPRO	SAMSN1	SLC52A1	TCAP
PTTG1	SASH3	SLC5A11	TESPA1
PYCARD	SCN4A	SLC6A13	TFAP2A
PYGM	SEC14L2	SLC7A7	TFAP2E
PYROXD2	SEL1L3	SLCO1A2	TFEC
QSOX1	SELE	SLCO1B3	TG
QSOX1	SELENOM	SLFN13	TGFB1
RAB34	SELPLG	SMAD5-AS1	THEMIS
RAC2	SEMA3C	SMIM32	THRSP
RAD54L2	SEMA4A	SMPDL3B	TIFAB
RAG1	SEMA6A	SNAP25	TIGIT
RANBP3L	SERPINE2	SNX20	TIMP1
RASAL1	SFN	SOAT2	TIMP4
RASGEF1A	SH2D1A	SOBP	TLDC2
RASGRF1	SH2D2A	SORCS2	TLR10
RASGRP1	SH2D5	SOWAHD	TLR7
RASL10B	SH3BP1	SOX4	TLR8

TM4SF19	UNC13D
TMC5	UPP2
TMC6	VANGL2
TMEM100	VAT1L
TMEM121B	VAV1
TMEM132A	VEGFD
TMEM150C	VNN2
TMEM156	VNN3
TMEM273	VSIG10L
TMEM51	VSTM4
TMEM51-AS1	WDFY4
TMPRSS3	WDR62
TMSB10/TMSB4X	WNK2
TNF	WNT10A
TNFRSF18	WNT4
TNFRSF8	YPEL1
TNFSF12-TNFSF13	ZAP70
TNFSF13B	ZBED2
TPPP2	ZBP1
TPRG1	ZIC5
TPTE2P1	ZNF280B
TREM1	ZNF296
TREM2	ZNF385B
TRIM46	ZNF385D
TRIM58	ZNF683
TRIM6	ZNF831
TRNP1	
TRPC4	
TRPM2	
TRPM3	
TRPV4	
TSPAN10	
TSPAN15	
TSPAN5	
TTC36	
TTLL4	
TXLNB	
TYMP	
UBASH3A	
UBASH3B	
UBD	
UBE2QL1	
UBXN10	
UGT1A1	
UGT1A3	
UGT1A4	
UHRF1	

Supplemental Table 2 : List of the 872 genes identified by RNAseq based on RelB mRNA expression. (related to Figure 2D).
