# Supplementary material for: The alternative NF-κB RelB subunit is a novel critical sensor of lipid metabolism to promote tumor development
Source: Cell Death Dis. 2026 Jul 4;17(1):830. doi: 10.1038/s41419-026-09064-7 (PMC13612402; doi:10.1038/s41419-026-09064-7)
Supplement: Supplementary file 7 [file 41419_2026_9064_MOESM7_ESM.pdf]

PNPLA8	CES3	GDPD1
PLCG2	PLD3	STXBP1
PNPLA7	APOC3	PLA2G6
LIPC	PRKN	LIPH
PLA2R1	PNPLA2	ANGPTL4
PLCD1	PLCH1	LMNB1
PRDX6	ARF1	APOA2
PLA2G4A	APOB	LIPG
PLB1	PRPAP1	PLA2G7
ENPP2	AGT	PRKCZ

Supplemental Table 3: List of 30 lipases and lipase-related genes identified by RNAseq based on RelB expression (related to Figure 3C).

Supplemental Table 3 *: Becquard, T.*, Benatar, C.* et al.*
